# Deamidation drives molecular aging of the SARS-CoV-2 spike protein receptor-binding motif

**DOI:** 10.1016/j.jbc.2021.101175

**Published:** 2021-09-07

**Authors:** Ramiro Lorenzo, Lucas A. Defelipe, Lucio Aliperti, Stephan Niebling, Tânia F. Custódio, Christian Löw, Jennifer J. Schwarz, Kim Remans, Patricio O. Craig, Lisandro H. Otero, Sebastián Klinke, María García-Alai, Ignacio E. Sánchez, Leonardo G. Alonso

**Affiliations:** 1 Centro de Investigación Veterinaria de Tandil (CIVETAN), CONICET-CICPBA-UNCPBA, Facultad de Ciencias Veterinarias, Universidad Nacional del Centro (FCV-UNCPBA), Tandil, Argentina; 2 European Molecular Biology Laboratory, Hamburg Unit, Hamburg, Germany; 3 Laboratorio de Fisiología de Proteínas, Instituto de Química Biológica de la Facultad de Ciencias Exactas y Naturales (IQUIBICEN), Facultad de Ciencias Exactas y Naturales, Universidad de Buenos Aires, Consejo Nacional de Investigaciones Científicas y Técnicas, Buenos Aires, Argentina; 4 Centre for Structural Systems Biology, Hamburg, Germany; 5 European Molecular Biology Laboratory, Heidelberg, Germany; 6 Departamento de Química Biológica, Facultad de Ciencias Exactas y Naturales, Universidad de Buenos Aires, Buenos Aires, Argentina; 7 Instituto de Química Biológica de la Facultad de Ciencias Exactas y Naturales (IQUIBICEN), CONICET-Universidad de Buenos Aires, Buenos Aires, Argentina; 8 Fundación Instituto Leloir, IIBBA–CONICET, and Plataforma Argentina de Biología Estructural y Metabolómica PLABEM, Buenos Aires, Argentina; 9 Instituto de Nanobiotecnologıa (NANOBIOTEC), UBA-CONICET-Universidad de Buenos Aires, Buenos Aires, Argentina

**Keywords:** SARS-CoV-2, receptor binding, spike, molecular aging, protein deamidation, RNA virus, protein evolution, protein–protein interaction, receptor structure–function, CoV, coronavirus, hACE2, human angiotensin-converting enzyme 2, PTM, posttranslational modification, RASA, relative accessible surface area, RBD, receptor-binding domain, RBM, receptor-binding motif, RBR, receptor binding ridge, SARS, severe acute respiratory syndrome

## Abstract

The spike protein is the main protein component of the SARS-CoV-2 virion surface. The spike receptor-binding motif mediates recognition of the human angiotensin-converting enzyme 2 receptor, a critical step in infection, and is the preferential target for spike-neutralizing antibodies. Posttranslational modifications of the spike receptor-binding motif have been shown to modulate viral infectivity and host immune response, but these modifications are still being explored. Here we studied asparagine deamidation of the spike protein, a spontaneous event that leads to the appearance of aspartic and isoaspartic residues, which affect both the protein backbone and its charge. We used computational prediction and biochemical experiments to identify five deamidation hotspots in the SARS-CoV-2 spike protein. Asparagine residues 481 and 501 in the receptor-binding motif deamidate with a half-life of 16.5 and 123 days at 37 °C, respectively. Deamidation is significantly slowed at 4 °C, indicating a strong dependence of spike protein molecular aging on environmental conditions. Deamidation of the spike receptor-binding motif decreases the equilibrium constant for binding to the human angiotensin-converting enzyme 2 receptor more than 3.5-fold, yet its high conservation pattern suggests some positive effect on viral fitness. We propose a model for deamidation of the full SARS-CoV-2 virion illustrating how deamidation of the spike receptor-binding motif could lead to the accumulation on the virion surface of a nonnegligible chemically diverse spike population in a timescale of days. Our findings provide a potential mechanism for molecular aging of the spike protein with significant consequences for understanding virus infectivity and vaccine development.

In December 2019, a viral pneumonia outbreak was reported in Wuhan, China (1). This outbreak quickly turned into a pandemic disease (COVID-19) of international concerns (https://covid19.who.int), and the novel pathogen causative of a severe acute respiratory syndrome (SARS) was soon identified as SARS-CoV-2, a new member of the *Betacoronavirus* genera. SARS-CoV-2 and SARS-CoV, the agents responsible for the 2002–2003 pneumonia outbreak, are closely related to the bat coronaviruses from which they likely originated and passed to an intermediate species that ultimately infected humans (2, 3). The host specificity and infectivity of SARS-CoV-2 and SARS-CoV rely on the spike protein (S). Through its receptor-binding domain (RBD, residues 319 to ∼515), S recognizes the human angiotensin-converting enzyme 2 (hACE2) with nanomolar affinity, triggering events that culminate with the fusion of the cellular and viral membranes (4). In SARS-CoV-2, the S protein is synthesized as a 1273-residue heavily glycosylated polypeptide that is cleaved by the host furin protease between the S1 (1–685) and S2 (686–1273) subunits (5). On the surface of native viruses, the S protein is mainly observed as a metastable trimer in the prefusion conformation. The RBD of each S protomer can switch between a receptor-accessible conformation known as the “up-state” and a receptor-inaccessible and buried conformation that packs against the N-terminal domain of the neighboring protomer called the “down-state” (6). Two regions can be identified in the RBD, a conserved core and a more variable region termed as the receptor-binding motif (RBM, residues 438–506). The latter region contains residues that establish direct contact with hACE2, determining S protein affinity and specificity (7).

Interspecies spillover is often observed in the coronavirus family members, a phenomenon that mainly originates from amino acid mutations in the RBD that enables S to bind ACE proteins from two different host species (8, 9). Beyond S crucial role in restricting viral host infectivity, the protein is the target of potent neutralizing antibodies (10, 11, 12) with therapeutic use and the main antigenic component of vaccines (13, 14). It is of particular interest to understand how mutations and posttranslational modifications (PTMs) in RBD affect viral infectivity, generate antigenic escape variants, or restrict the humoral and cellular immunity.

Asparagine (Asn) deamidation is a frequently observed spontaneous and irreversible PTM (15, 16). As a result of the substitution in the Asn side chain of the carboxamide nitrogen atom by a hydroxyl group, a mixture of aspartic and isoaspartic acid (a beta amino acid) is generated (17), introducing a negative charge and a rearrangement of the protein backbone in the latter case. The deamidation rate, which heavily depends on the primary sequence and local structure, can be estimated using bioinformatic tools that rely on different approaches such as structural constraints, machine learning, or primary sequence and disorder predictors (18, 19, 20). The fastest deamidation rate (half-time ∼1.2 days) is often observed for the asparagine–glycine (NG) dipeptide in loosely structured regions, which are generally regarded as deamidation "hotspots" for which most of the methods provide accurate prediction (21). In this regard, although asparagine deamidation is a relatively slow process (half-times of hours to days), it can significantly occur within the time frame in which some critical viral processes develop. For instance, the SARS-CoV-2 virus can remain infective for a couple of days after incubation at 37 °C (22).

The S protein contains multiple deamidation sites, but only some of them bear biological relevance. To identify these relevant sites, we applied a prioritization process based on four key points: (i) *in silico* identification of hotspots and deamidation rates using the NGOME-LITE algorithm (23), (ii) conservation of the sites among *Betacoronavirus*, (iii) location of the sites at the ACE2-binding surface, and (iv) experimental determination of the deamidation rates at specific hotspots by mass spectrometry.

A direct consequence of deamidation events in Asn residues located at the RBM is the modification of the electrostatic potential of the receptor-binding surface due to the introduction of a negative charge from the aspartic or isoaspartic carboxylate. Consequently, as the RBD is the preferential region from the S protein for neutralizing antibodies (10, 11, 12), deamidation could also affect the efficiency of the humoral immune response. With these data we generated a model that computes the proportion of deamidated protomers in a single viral particle and predicts how these species evolve. Our findings shed light on deamidation, an aging mechanism that operates on the RBM, a critical region that determines virus infectivity and may profoundly impact on vaccine development.

## Results

### Identification of deamidation hotspots in S proteins

**Figure 1:**
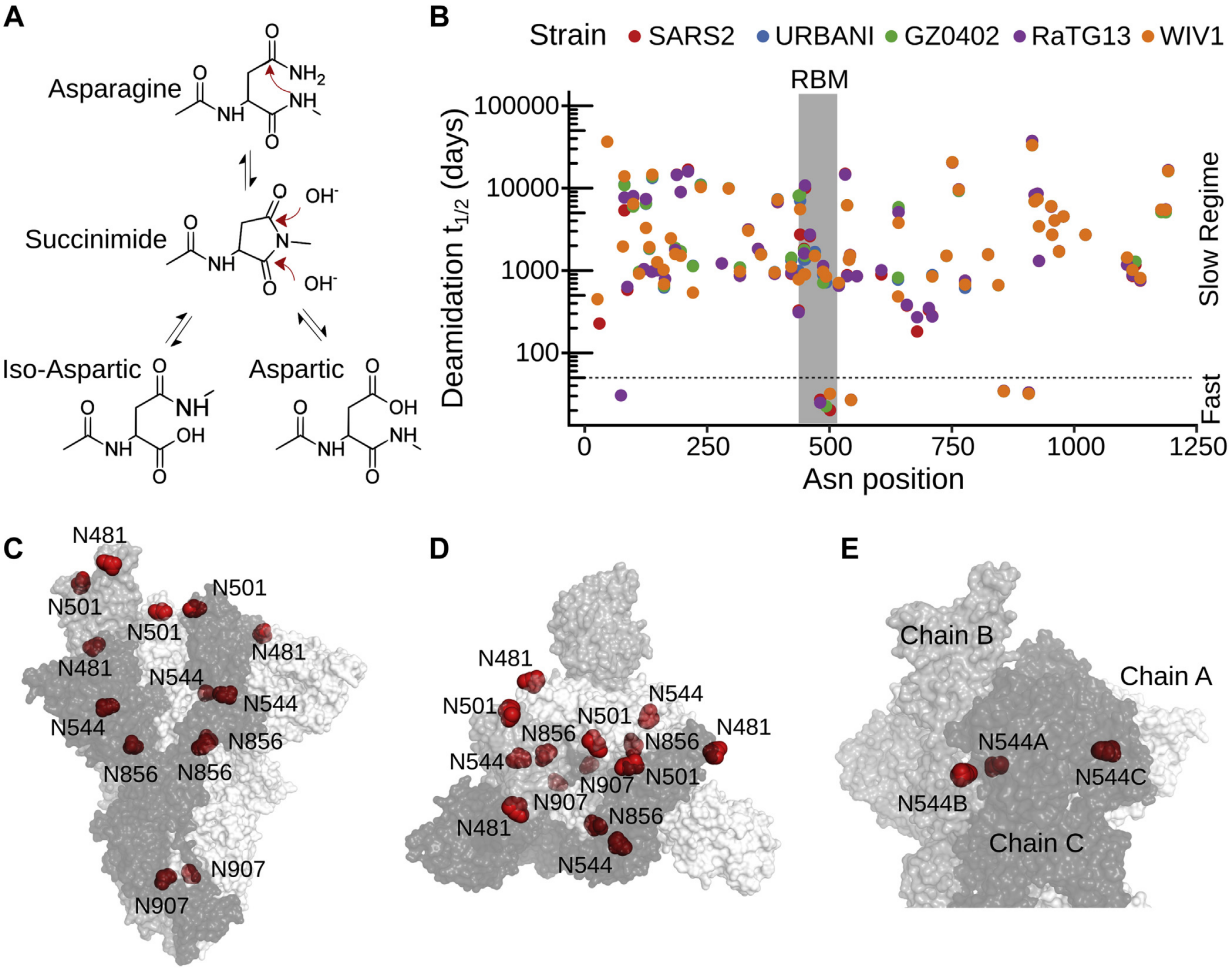
**Deamidation profile of S from *Betacoronavirus***. *A*, schematic representation of the deamidation reaction mechanism. *B*, deamidation profile of S proteins from a group of betacoronaviruses obtained with NGOME-LITE; only nonglycosylable asparagines are included. Deamidation half-times are shown on logarithmic scale. The Asn positions are referred to the SARS-CoV-2 sequence. *C*, deamidation hotspots on the SARS-CoV-2 S protein are shown in *red*. Protomers are colored in different *gray* tones. The PDB 6zgg (24) was used for this analysis, which contains a furin-cleaved SARS-CoV-2 S trimer, with two RBDs in the down conformation (chains A and C) and one in the up conformation (chain B). *D*, top view of (*C*), showing exposed deamidation hotspots at the RBD (Asn 481 and 501). *E*, zoom of the exposed Asn 544 residues.

We initially estimate the individual deamidation (Fig. 1*A*) half-time (t_1/2_) using the NGOME-LITE (23) algorithm, for all Asn residues with no glycosylation probability for a set of *Betacoronavirus* S proteins (Fig. 1*B*). We restricted our analysis to a group of S proteins from betacoronaviruses with a demonstrated affinity for the hACE2 receptor, which includes SARS-CoV-2 (Wuhan), SARS-CoV (Urbani), SARS-CoV (GZ0402), and the closely related Bat SARS-like CoVs RaTG13 (24) and WIV1 (25) (Table S1).

A similar deamidation profile is observed for the five S proteins (Fig. 1*B*), characterized by a major group of Asn residues in a slow-deamidation regime with estimated *t*_1/2_ greater than 100 days and a restricted group of residues in a fast-deamidation regime, with estimated *t*_1/2_ ranging between 20 and 35 days (Fig. 1*B* and Table S2). All Asn residues in the fast-deamidation regime (hereafter referred to as deamidation hotspots) are part of NG dipeptides (Fig. S1), for which a deamidation *t*_1/2_ of 1.2 days is expected in the absence of structural protection (*i.e.*, in a short nonstructured peptide) (17, 18), indicating that the S protein fold protects these Asn residues from deamidation. Slow regime residues virtually do not deamidate during the viral life cycle and are devoid of any functional consequence.

Except for the single deamidation hotspot observed at position 74 in the Bat-CoV RaTG13 (in SARS-CoV-2 Asn residue 74 is glycosylated (26)), deamidation hotspots are restricted to a group of residues clustered between residues 481 and 501 in the RBM, including residue 493 (only observed in the GZ0402 SARS-CoV), which shows partial conservation among analyzed CoVs, and a group of strictly conserved residues at positions 544, 856, and 907 (Figs. 1*C* and S1), observed in all the selected S sequences. Hotspots within the RBM are, altogether, termed as the *RBM deamidation cluster*. Overall, the deamidation profile shows that hotspots are not evenly distributed along with these S protein sequences (Fig. 1, *B* and *C*).

Beyond sequence context, the protein fold, backbone flexibility, and surface accessibility correlate with the experimental Asn deamidation rates (27, 28). The hydrolysis of the cyclic intermediate is required to complete the deamidation reaction (Fig. 1*A*) for which solvent molecules must reach the reaction center. Solvent accessibility can be assessed by calculating the relative accessible surface area (RASA) of the deamidation-prone residue to a small probe radius of 1.4 Å (a water molecule). In addition, NGOME-LITE cannot sense the effect of the tertiary and quaternary structure on the deamidation rate, factors that become of critical importance for hotspots centered at the interdomain or interprotomeric interfaces of the S trimer. Higher-order structure factors affecting deamidation rates can be identified by evaluating residue accessibility to a 3.0-Å radius probe.

To discriminate between the predicted deamidation-prone residues that are solvent accessible and those present at the interdomain surface we calculated the side-chain RASA for all deamidation hotspots in the SARS-CoV-2 S structure. We performed our analysis using a structure of the furin-cleaved S protein in the prefusion conformation, which contains two RBDs in the down-state and one in the up-state (PDB 6zgg (24) using two probes with radii of 1.4 and 3.0 Å (29), Table S3).

All five deamidation hotspots are fully or partially accessible to the 1.4-Å radius probe (Table S3), albeit to a different extent depending on the RBD conformation, indicating that they are accessible to water molecules, a requirement for hydrolysis. On the other hand, only hotspots 481 and 501 are accessible to the 3.0-Å radius probe in both the up and down RBD conformations (RASA of 74.2% and 25.8% in the up conformation for Asn 481 and 501, respectively), (Fig. 1*D*) whereas Asn 544 is virtually buried in the RBD in the down-state and partially exposed (RASA of 25.6%) in the up-state (Fig. 1*E*).

In addition, Asn 907 located in the heavily glycosylated stalk of S is partially accessible to the 1.4-Å radius probe through an internal channel formed by the three protomers, whereas Asn 856 is packed between the contact interface of two protomers (Fig. 1*C*). Both positions are fully inaccessible to the 3.0-Å radius probe, irrespective of the RBD conformation.

### Assessment of deamidation *t*_1/2_ for the 481, 501, and 544 hotspots in mild conditions

The deamidation profile of SARS-CoV-2 S obtained with NGOME-LITE shows the presence of conserved deamidation hotspots at relevant protein–protein recognition interfaces. However, the fact that these sequence stretches are part of a large multidomain protein, which is not detected by the NGOME-LITE algorithm, reduces the prediction accuracy of this bioinformatic tool.

The first experimental observation of the occurrence of deamidation at the predicted RBD hotspots was reported previously by the Wells laboratory (26). To monitor N-glycan occupancy in the full-length S protein, they observed 18.9% of ^18^O-Asp conversion at Asn 501, 4.8% at Asn 481, and 7.8% at Asn 544 attributable to deamidation, since these hotspots lack canonical glycosylation sequons (N-X-S/T) and were shown to have less than 5% of glycan occupancy. The amount of ^18^O-Asp conversion correlates well with the deamidation *t*_1/2_ predicted by NGOME-LITE for all five deamidation hotspots (Table S4).

As the deamidation kinetics is critical to evaluate the potential effect of the aspartic/isoaspartic formation in the S function, we experimentally assessed the deamidation *t*_1/2_ for the hotspots 481, 501, and 544 in a recombinantly expressed extended version of the SARS-CoV-2 RBD construct encompassing residues 319 to 566 (30) at 4 and 37 °C.

**Figure 2:**
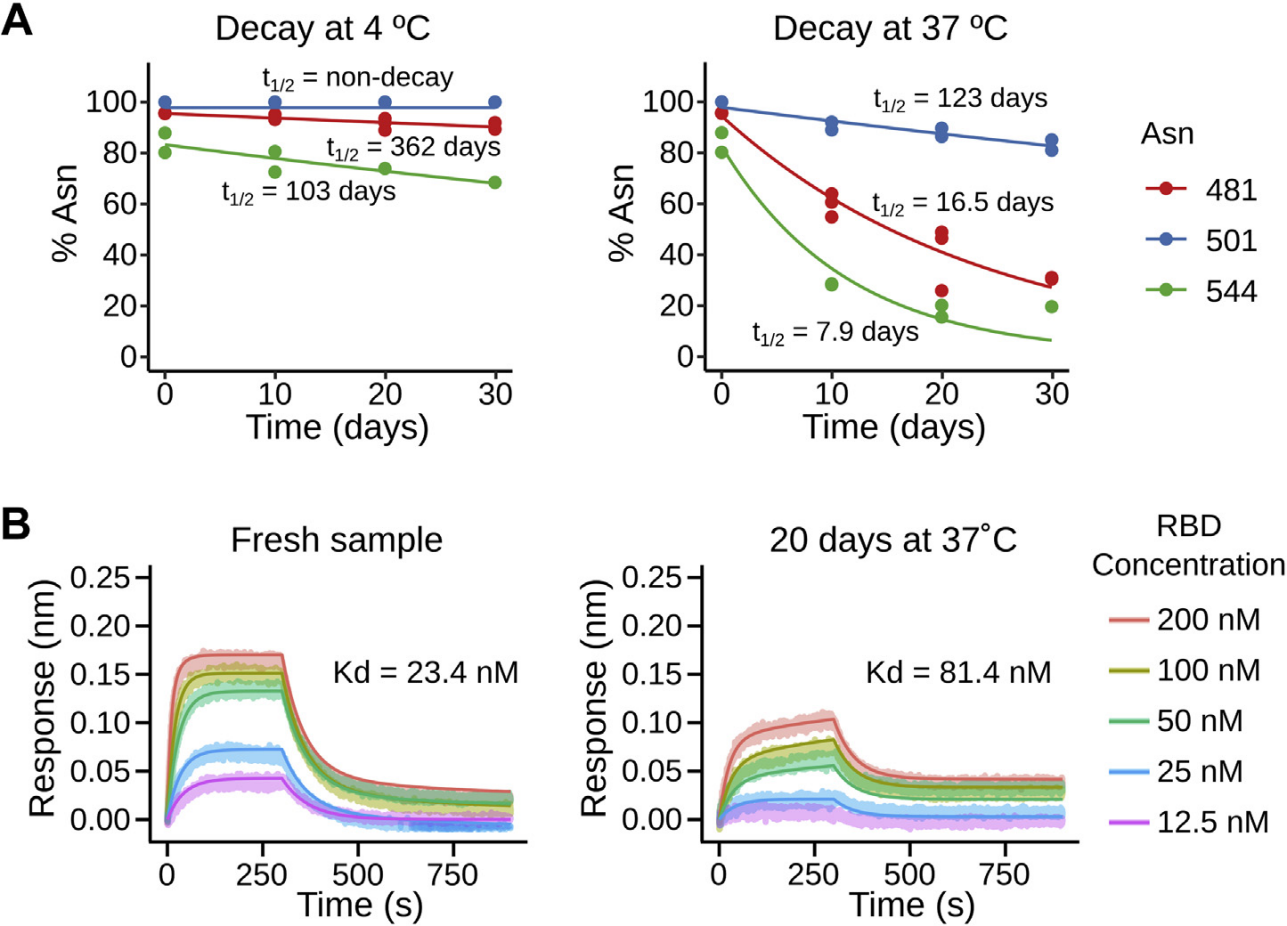
**Experimental deamidation kinetic and affinity to hACE2 of an aged RBD sample**. *A*, *left*, time-decay of asparagine-containing peptides for the 481, 501, and 544 hotspots at 4 °C. The lines are fit to an exponential function with initial amplitude of 100% and endpoint of 0%. *Right*, time-decay of asparagine-containing peptides for the 481, 501, and 544 hotspots at 37 °C. The lines are fitted to an exponential decay. *B*, *left*, biolayer interferometry response curves of a fresh RBD sample to an immobilized hACE2. *Right*, biolayer interferometry response curve of an aged RBD sample (20 days at 37 °C). RBD, receptor-binding domain.

It should be noted that residue 544 is not part of the RBD, but instead it belongs to the structurally conserved subdomain 1 that encompasses residues ∼516 to ∼591 not fully present in our construct and the subdomain 1 may be devoid of its native structure. Consequently, the *t*_1/2_ for the 544 hotspot is not further considered for functional conclusions. However, it is shown as a corroboration that the technique is adequate for determining deamidation half-times in the expected time lapse. In our experimental setup, the recombinant RBD protein was incubated at pH 7.4 over several days at two different temperatures and the presence of deamidated species was identified and quantified by mass spectrometry (31) (Fig. S2 and Table S5). Figure 2*A* shows the time-decay of the unmodified peptides (Asn containing peptides) bearing the deamidation hotspot 481, 501, and 544. Full data are available *via* ProteomeXchange with identifiers PXD028071 (peptide: IYQAGSTPCNGVE) Dataset S1 and PXD027873 (peptides: CVNFNFNGLTGTGVLTEand GFNCYFPLQSYGFQPTNGVGYQPYR) Dataset S2.

Initially (time = 0), the unmodified peptides covering the 481, 501, and 544 hotspots account for 95.6%, 100.0%, and 84.0% of the detected peptides, respectively (Fig. 2*A* and Table S4); these values dropped to 34.9%, 87.9%, and 17.4% for the 481, 501, and 544 hotspots, after 20 days of incubation at 37 °C.

Deamidation half-times of 16.5 ± 3.7, 123 ± 23, and 7.9 ± 1.2 days were obtained at 37 °C for the 481, 501, and 544 hotspots in the SARS CoV-2 RBD protein. Under our experimental conditions, the RBD hotspot 501 remains stable against the deamidation process despite being identified *a priori* by the NGOME-LITE as the fastest deamidation hotspot. On the contrary, Asn 544 has the highest deamidation rate, with a value close to the expected deamidation rate of an NG sequence in an unstructured peptide model (1.2 days), supporting that this residue is placed in an unstructured region. The disagreement between experimentally determined deamidation rates and computational predictions for hotspots that share the common NG sequence highlights that protein structure and associated dynamics in S can fine-tune the deamidation rate.

As expected, the deamidation reaction was slowed down considerably by decreasing the temperature to 4 °C (Table S4). The deamidation half-time for Asn 481 at 37 °C compared with 4 °C is more than 20-fold higher, highlighting that temperature can critically affect the identity of the RBM.

Deamidated species for hotspots 481 and 544 elute at two different retention times in a reverse-phase chromatography experiment (Fig. S2), in agreement with the reaction mechanism that proposes the generation of a mixture of aspartic or isoaspartic bearing peptides. In reverse-phase chromatography, deamidated species containing isoaspartic residues are reported to elute faster than aspartic-containing peptides (17), and based on this chromatographic behavior, the deamidation reaction (20 days at 37 °C) at hotspots 481 and 544 shows an abundance of 46.6% and 68.5% of isoaspartic containing species, respectively (Table S4). On the contrary, only one peak was observed for the deamidated species at Asn 501.

We then evaluated how deamidation affects the affinity of RBD for the ectodomain of hACE2. To this end we performed a biolayer interferometry assay using an aged (20 days at 37 °C) and heterogeneously deamidated RBD sample with ∼65%, ∼12%, and ∼83% of hotspots 481, 501, and 544 in its deamidated form. We observed that the unaged RBD binds hACE2 with an affinity dissociation constant (K_d_) of 23.4 ± 0.8 nM, whereas the aged RBD has a reduced affinity of only 81.4 ± 0.8 nM (Fig. 2*B*) showing that deamidation is detrimental for hACE2 binding and might impact virus infectivity.

To put this result into context, we must consider that the aged protein is a heterogeneous mixture of different chemical species, where, in a single RBD molecule, different combinations of nondeamidated and deamidated hotspot in their aspartic or isoaspartic forms coexist. Assessing how each chemical species (each individual hotspot in its aspartic or isoaspartic form) contributes to hACE2 binding is a nontrivial experimental task. The major drawback is the fact that deamination mainly introduces an isoaspartic residue into the hotspot, which produces, beyond the charge effect, a significant rearrangement of the protein backbone, making it difficult to emulate it faithfully by a direct aspartic mutation. Likewise, and although deamidation is the main posttranslational modification observed in the aged protein, other less frequent PTMs (such as methionine oxidation) could contribute, in some proportion, to the decrease in the affinity for hACE2.

On the other hand, deep mutational scanning experiment performed by the Bloom laboratory (32) describes the effect of individual aspartic mutations at positions 481 and 501. This experiment shows a marginal perturbation in the apparent dissociation constants (K_D,app_) of the RBD–hACE2 complex for the N481D mutation (Δlog10K_D,app_ -0.07 ± 0.01; K_D,app_ Mut/K_D,app_ Wild type ∼1.2-fold) but a significant decrease in the interaction strength (Δlog10K_D,app_ −2.42 ± 0.03; K_D,app_ Mut/K_D,app_ Wild type ∼260-fold) for the N501D mutation (32). We observed a 3.5-fold decrease in the K_d_ for an aged RBD sample containing ∼65% of deamidated hotspot 481, harboring mostly an isoaspartic residue (∼47%, Table S4), and ∼12% of deamidated hotspot 501 harboring only an aspartic residue (Table S4). Taking these results together it is likely that deamidation at each RBM hotspot additively affects hACE2 binding, with an overwhelming importance of 501 position.

### Topological constraints drive localization of conserved deamidation hotspots at the RBM

**Figure 3:**
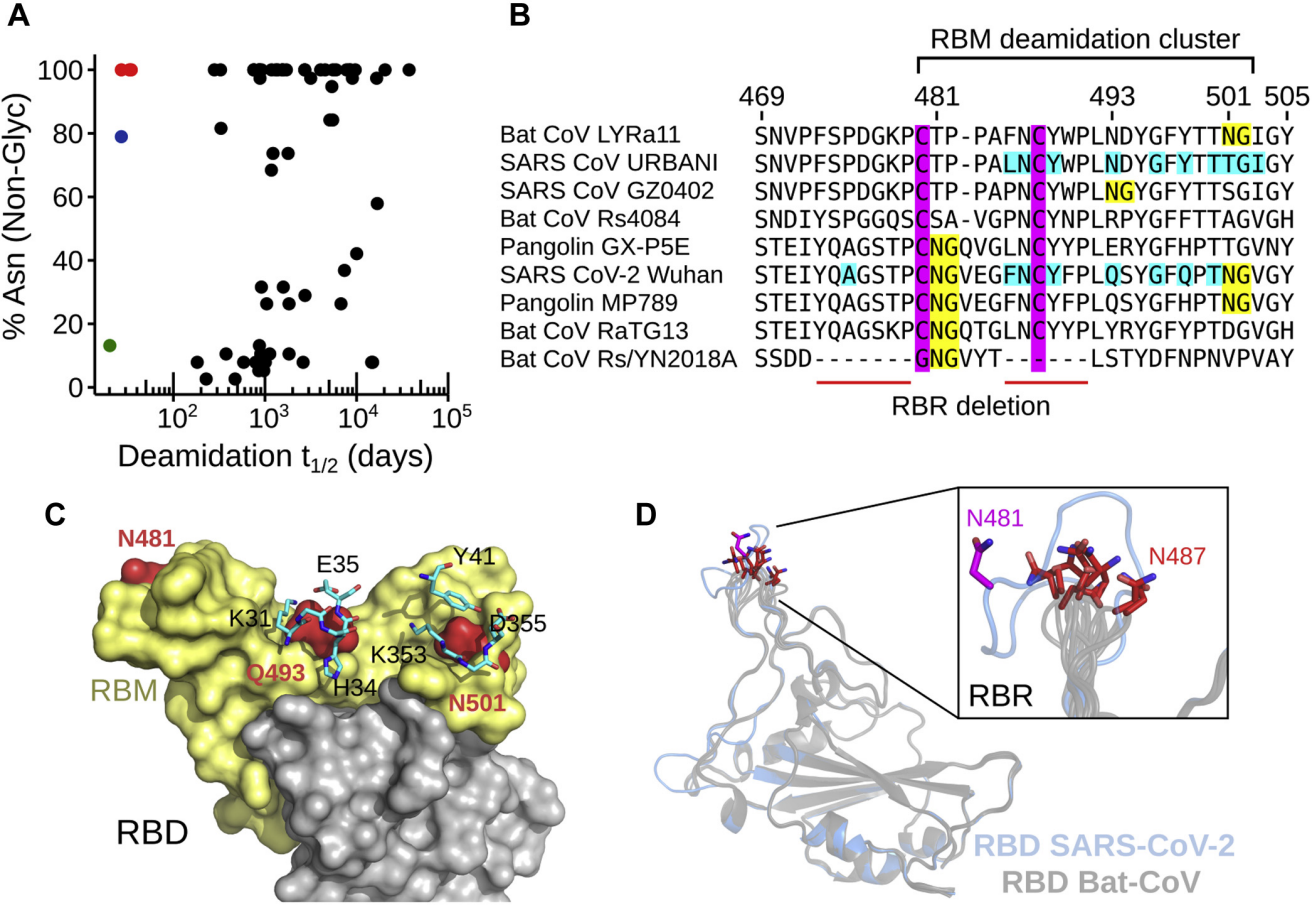
**Conservation pattern of deamidation hotspots at the RBM of Sarbecoviruses**. *A*, conservation of asparagines in SARS-Cov-2 shown as the percentage of Asn present in 38 sarbecoviruses *versus* deamidation half-times (Dataset S3). Hotspots 544, 856, and 907, *red dots*; hotspot 481, *blue dot*; and hotspot 501, *green dot*. *B*, alignment of different S proteins from selected sarbecoviruses. The RBM region is indicated, and the deamidation hotspots are highlighted in *yellow*. Residues of SARS-CoV and SARS-CoV-2 located closer than 5 Å from hACE2 residues are highlighted in *cyan*. *C*, location of the deamidation hotspots 481, 493, and 501 at the surface of SARS-CoV-2 RBD (pdb: 6M0J). Deamidation hotspots are shown in *red*, RBM (residues 438–506) is shown in *ocher*, and the core of the RBD is depicted in *gray*. hACE2 residues that directly interact with the RBM deamidation hotspots 493 (K31, H34, and E35) and 501 (Y41, K353, and D355) are shown as *sticks*. In SARS-CoV-2, the position 493 is a glutamine. *D*, superposition of RBD from SARS-CoV-2 (pdb 6M0J, *pale blue*) and an ensemble of modeled structures of RBD from bat-CoV Rs/YN2018A (*gray*). Asn 481 residue in SARS-CoV-2 RBD is shown in *magenta sticks*, and the Asn residues corresponding to the predicted 487 deamidation hotspot in Rs/YN2018A are drawn in *red sticks*. The *inset* details the receptor binding ridge (RBR) region. RBD, receptor-binding domain; RBM, receptor-binding motif.

Owing to the critical role of the RBM residues in determining hACE2 affinity and host specificity (33, 34), we further evaluated the conservation pattern of hotspots at the RBM deamidation cluster by extending our analysis to a group of 38 betacoronaviruses representative of the subgenus *Sarbecovirus* (35, 36), which, in addition to SARS-CoV and SARS-CoV-2, includes numerous bat and pangolin viruses (Fig. 3*A*, Dataset S3, File S1 and Table S6). We evaluated the correlation between Asn conservation among the selected *Sarbecovirus versus* the estimated deamidation half-time of Asn residues in the SARS CoV-2 S protein, and the results are highlighted in Figure 3*A*.

Overall, the RBM deamidation cluster shows a variable conservation pattern (Fig. 3, *A* and *B*) compared with hotspots 544, 856, and 907, which are identified as red dots in the left upper region of Figure 3*A*, indicating that these Asn are both conserved and deamidation-prone residues. On the other hand, hotspots within the RBM deamidation cluster are differentiated based on their opposite conservation pattern. The hotspot 493 occurs only once in the SARS-CoV GZ0402, and hotspot 501, identified as a green dot in the Figure 3*A*, shows low conservation (observed in 5 of 38 sequences) and hotspot 481 (blue dot in Fig. 3*A*) is highly conserved and present in 30 of 38 sequences (∼80% conservation).

Moreover, the RBMs of the *Sarbecovirus* members can be differentiated into two groups according to the presence of a *receptor binding ridge* (RBR), which involves residues ∼470 to 488 and includes a disulfide bridge between Cys 480 and 488 (36), highlighted in purple in Figure 3*B*. Altogether, deamidation hotspots in the RBMs containing an RBR are observed within an 11-amino-acid stretch and are restricted to any of the following three positions: 481, 493, and 501 (Fig. 3*B*). The Bat CoVRs/YN2018A, included as a representative sarbecovirus that lacks an RBR and does not bind hACE2, shows a predicted deamidation hotspot at position 487 (Table S7) that cannot be easily aligned with the RBR-bearing sequences (Fig. 3*B*). However, the superposition of the RBDs from SARS-CoV-2 (pdb: 6M0J) and Bat CoVRs/YN2018A obtained by homology modeling shows that the 481 and 487 hotspots are located in a similar position in the three-dimensional structure of the domain enabling sequence alignment between both groups. This suggests that topology drives conservation of deamidation-prone Asn residues at this specific position (Fig. 3*D*).

The overall folds of SARS-CoV and SARS-CoV-2 RBMs are similar with 14 residues (34) observed in equivalent positions in both structures located at less than 5 Å from hACE2-binding residues (cutoff for considering a direct contact) including the 493 and 501 deamidation hotspots (Fig. 3*B*). Asn 493 and 501 form a direct interaction with hACE2 residues K31 and K353, respectively, which are considered critical for S binding. Mutational studies (33, 34, 37) have shown the relevance of these two positions in determining hACE2 affinity. In particular, the presence of aspartic acid or asparagine at position 501 in the spike protein determines the possibility of infecting cells through interaction with hACE2. The RaTG13 CoV and SARS CoV-2 share 89.2% of identity at the RBD; however, the RaTG13 spike protein does not enable an efficient hACE2-mediated infection in a pseudovirus assay (38, 39). Of the six different positions between RaTG13 and SARS CoV-2 that most contribute to the hACE2 binding (Y449, F486, Q493, Q498, N501, and Y505 in SARS CoV-2), the most important is the aspartic acid at position 501 in the RaTG13 RBD. A single D501N mutation in the RaTG13 increases 9-fold the affinity for hACE2 (38) and restores infectivity in a cell–cell fusion assay (39) pinpointing the biological importance of this deamidation hotspot.

On the other hand, the deamidation hotspot at position 481 is fully exposed in the RBD, positioned in the external wall of the RBR (Fig. 3, *C* and *D*), and does not directly participate in hACE2 binding.

Taking together the sequence and topological conservation patterns and the measured deamidation half-times we conclude that deamidation hotspots at the RBM are subjected to different selection forces. The hotspots 493 and 501, which significantly contribute to hACE2 binding, are not highly conserved among sarbecoviruses and are not significantly deamidated under our experimental conditions. The occurrence of a deamidation event at these hotspots would be highly detrimental for receptor binding. Furthermore, and of note, the N501Y mutation that eliminates the deamidation hotspot is a hallmark of the emerging SARS-CoV-2 alpha (VOC 202012/01, B.1.1.7) and beta (501Y.V2, B.1.351) variants that are rapidly spreading in the United Kingdom and South Africa (https://www.who.int/csr/don/31-december-2020-sars-cov2-variants/en/). Both variants are likely to have arisen independently and are associated with increased transmissibility.

A different mechanism might have shaped the presence of a deamidation hotspot at position 481 or equivalent positions that are topologically conserved beyond the absence of an entire structural element (the RBR) and that readily deamidated under physiologically relevant conditions. Although deamidation is often conceived as detrimental for protein function or stability, our findings support the striking possibility that deamidation mediates an aging-dependent gain of function in S protein.

### Kinetic model for S protein deamidation in the SARS-CoV-2 virion

As spontaneous Asn deamidation is, compared with other modifications that regulate protein function, a slow process, we focused on finding to what extent deamidation of the S protein RBM takes place in the context of the SARS-CoV2 life cycle. We have contextualized the results obtained for Asn 481 and 501 by numerical simulation and graphical representation of the SARS-CoV2 virion. We used the Gillespie algorithm as implemented in COPASI (40) to simulate the stepwise irreversible transition of the 33 S protein trimers in a virion (41, 42) from the intact state to the fully deamidated state. Our model considers deamidation of only Asn 481 and 501, with independent experimental half-time at 37 °C (Fig. 2). The six deamidation sites in each S trimer lead to 2^6^ possible deamidation states, which can be grouped into 20 species using symmetry considerations (Fig. S3).

**Figure 4:**
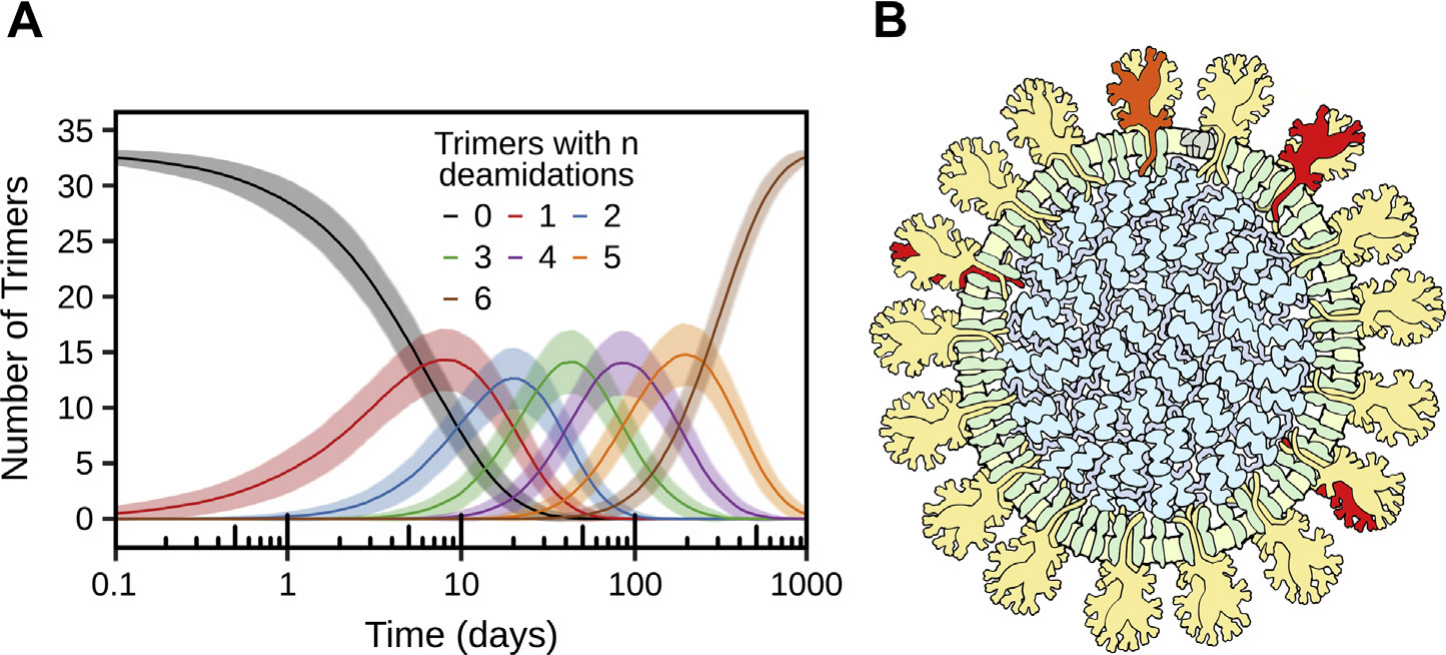
**Spike protein hotspot deamidation in the context of the SARS-CoV2 virion at 37 °C**. *A*, simulated time course of the number of trimers with zero to six deamidation events at sites Asn 481 and Asn 501, using the deamidation half-times from Figure 2 (Fig. S3, for simulation details). We report the average and standard deviation of 1000 simulations using the Gillespie algorithm (Fig. S4 for full results and Table S8 for the results at several time points of interest). *B*, visualization of S protein deamidation in the SARS-CoV2 virion 48 h after synthesis. A cross section of the virus is shown (see (22) for details), with membrane and membrane-bound viral proteins M and E in green and the viral genome and associated nucleocapsid proteins N in *blue*. A total of 17 of the 33 spike trimers are shown, colored according to the simulation results (Table S8). *Yellow*, undeamidated spike monomers. *Red*, spike monomers deamidated at Asn 481. *Orange*, spike monomers deamidated at Asn 501.

We performed 1000 stochastic simulations and reported the average and standard deviation of the results for each time point. Figure S4 shows the full results, whereas Table S8 highlights the results at several time points of interest. The evolution of the different deamidated species of Spike trimers present in a virion at 37 °C is shown in Figure 4*A*, grouped by the total number of deamidated sites in the trimer for clarity. The intact S trimers decay with a half-time of approximately 4 days. This value is heavily affected by temperature, and the intact S trimer t_1/2_ decay at 4 °C is delayed to 110 days. Owing to the multimeric nature of both the virion and the Spike protein, trimers with at least one deamidated hotspot increase early: after only 1 day, close to four S trimers would host a deamidated hotspot (mainly at Asn 481). After 2 days, close to seven of the S trimers have deamidated at one site and close to an additional trimer has deamidated at two sites (Table S8). Figure 4*B* shows a visualization of the deamidation state of the SARS-CoV2 virion after 48 h (43). Only 17 S protein trimers are shown for clarity (43), whereas the population of each species is proportional to that in Table S8.

Our model supports the existence of a nonnegligible population of intravirion S protomers bearing a deamidated hotspot 481 that accumulate within 24 to 48 h, a time-lapse compatible with the viral life cycle (*i.e.*, the period in which SARS CoV-2 remains infective when incubated at 37 °C (22)). Of note, the molecular mechanism that enables the emergence of intravirion deamidated S proteins is conserved among *Sarbecovirus* species and located at topologically equivalent positions in the different RBDs. In addition, it is important to note that deamidation may have significantly altered the surface properties of the virion only 2 days after synthesis and this may have consequences for immune evasion.

On the other hand, the intact S trimers significantly decrease to four after 2 weeks, whereas the full deamidation of the virion takes about 1000 days. This timescale is far slower than that of SARS CoV-2 inactivation in solution at 37 °C (22), indicating that deamidation of RBM hotspots might not be the main molecular mechanism for viral inactivation.

## Discussion

Asn deamidation is a PTM that spontaneously and pervasively occurs in proteins containing the dipeptide sequence NG. The quantitative assessment of the time-dependent accumulation of deamidated species at specific positions in large and heavily posttranslationally modified proteins is a difficult experimental task, and consequently, its potential functional role has been neglected. Deamidation is often conceived, by default, as a degradative reaction that is detrimental for protein function and stability. Exceptionally, deamidation gains importance in biologics and vaccine development when deamidating-prone Asn residues are located, by chance, in protein–protein binding interfaces or in antigenically relevant epitopes that critically affect protein performance (44, 45, 46). With the exception of a handful of well-documented cases (47, 48), the participation of Asn deamidation as a mechanism that ultimately leads to a time-dependent protein gain of function is scarce. Here we present biochemical and bioinformatics evidence that (i) deamidation hotspots are a conserved trait in the RBM of *Sarbecovirus*, (ii) the measured deamidation half-times for individual Asn residues enable the accumulation of intravirion deamidated species during the virus life cycle, and (iii) conservation is driven by topological constraints. Taken together, these results suggest a yet unidentified functional role for conserved deamidation hotspots.

The deamidation profile of the S proteins from a selected group of betacoronaviruses shows a discrete number of predicted deamidation hotspots that are not randomly distributed over the entire length of the S ectodomain. Instead, deamidation hotspots are observed either fully conserved or clustered within the RBM. The RBM cluster is of particular importance owing to its role in hACE2 binding and includes hotspots at positions 481, 493, and 501 that are partially conserved among *Sarbecovirus*.

We experimentally observed that Asn 481 deamidates with a half-time of 16.5 days at 37 °C, a reaction that is highly temperature sensitive. An aged RBD sample in which 65% of the 481 hotspots is observed in its deamidated form showed a 3.5-fold decrease of the K_d_ for hACE2. However, the idea that deamidated residues are always detrimental for protein fitness collides with the observation that hotspot 481 is highly conserved among *Betacoronavirus*. Moreover, topological constraints are likely to drive the conservation of a deamidation hotspot at position 487, resembling the 481 position of SARS CoV-2, in S protein of related bat *Betacoronavirus* that have suffered a deletion of the entire RBR region, a critical element for hACE2 binding. Such topological constraints have been described to drive the evolution of reactive residues that regulate protein function (49).

On the other hand, deamidation hotspots in the RBM cluster that are critical for hACE2 binding (positions 493 and 501) show a low conservation profile among *Sarbecovirus*. The deamidation hotspot at position 501 is in direct contact with the K353 residue in hACE2 (34), and different observations reveal its critical role in determining hACE2 affinity (32, 50).The N501Y mutation that eliminates this deamidation hotspot is a hallmark of the emerging SARS-CoV-2 variants, such as the B.1.1.7 (UK variant), B.1.351 (South Africa variant) and P.1 (Brazil variant) (51). In our experimental setup, we did not find any significant deamidation of Asn 501 in the SARS CoV-2 RBD construct. Hotspots 493 and 501 are frequently mutated in naturally emerging variants of SARS-CoV or SARS-CoV-2, suggesting that they are susceptible to selective pressure, albeit the slow deamidation half-times experimentally observed for these positions suggest that deamidation would be neutral from an evolutive standpoint.

It should be noted that experimental evidence about deamidation of Asn 501 was reported previously for this protein (26). It is expected that discrepancies may arise in using of different experimental conditions that may affect protein conformation and, hence, the deamidation rate. Alternatively, deamidation at these sites might not occur in the native, well-folded S protein but might be rapidly triggered when the protein is proteolyzed during the antigenic presentation process, interfering with the host immune response (45).

Our integral kinetic model for the virion deamidation illustrates how the multimeric nature of S protein and the virion affects the accumulation of deamidate species. The model considers only two hotspots with their respective observed experimental deamidation half-times. The model shows that deamidation would not be the only molecular mechanism that reduces virus infectivity. The full deamidation time of the virion in which all of its 33 S trimers of the external surface are deamidated at both hotspots is close to 1000 days, far slower than the time required to eradicate the virus infectivity by the sole incubation at 37 °C, which has been reported to be a couple of days (22). However, partially deamidated species are readily accumulated within hours and after a day close to four trimers would host a deamidated protomer, mainly at Asn 481. This supports the possibility that deamidated hotspots can affect Spike fitness. For example, deamidation supports the coexistence of chemically diverse RBM populations (*i.e.*, with different primary sequence) within the virion, some of which may enable the virus to evade antibody recognition while its effect in receptor binding is moderate. In this line, the E484K mutation, which coexists with the N501Y in the Brazilian SARS CoV-2 P.1 lineage (51), located nearby to Asn 481 in the RBR, has been associated with antibody escape, suggesting that mutations or modifications in the RBR region may be related to an immune evasion viral mechanism (52, 53, 54).This let us speculate that conservation of deamidation hotspots in topologically equivalent positions pursues some functionality. It has been shown that deamidation of viral capsid proteins can significantly affect ligand recognition. Deamination of N373 in the Norovirus VP1 protein, which spontaneously occurs with a half-times of days yielding an isoaspartic residue, dramatically affects the recognition of histo blood group antigens, a critical step for Norovirus infection (55).

On the other hand, deamidation hotspot at position 493 (Asn 479 in the SARS-CoV sequence), which is in direct contact to K31 in hACE2 (8, 34), was only observed in SARS-CoV GZ0402, which has been isolated from a handful of individuals. Unlike the most common SARS-CoV Urbani that possesses the dipeptide Asn–Asp at positions 479 to 480, which is not expected to be a deamidation hotspot, the SARS-CoV GZ0402 bears an Asn–Gly hotspot. Of interest, a deamidation hotspot is generated in antibody escape mutants of SARS-CoV recovered under the selective pressure of the R80 neutralizing monoclonal antibody. The escape variants bear a D480G mutation that transforms an ND slow-deamidating Asn into an NG deamidation hotspot (56, 57).

Overall, our data show that deamidation hotspots are enriched and conserved in the RBD of *Sarbecovirus*, and SARS-CoV-2 in particular, suggesting that deamidation at specific positions might influence viral fitness. The nonnegligible accumulation of deamidated S protein within a virion during the viral life cycle supports the possibility that deamidation mediates some functionally relevant mechanism. However, it is unclear how such a slow, spontaneous, and irreversible PTM might affect viral biology. Deamidation at the conserved hotspot 481 in the RBR might induce a radical change in the charge and the backbone of the RBM enabling the virus to escape many neutralizing antibodies, or in addition, it might generate a noncanonical integrin-binding motif (48), triggering a new function in those deamidated protomers.

Beyond the identification of deamidation hotspots in the S protein of SARS-CoV-2, a previously neglected attribute with impact on the understanding of the biology of this highly infective virus, our combined rational is suitable for prioritizing the identification of any deamidation hotpots that, impacting fitness, have been evolutionarily conserved.

## Experimental procedures

### Deamidation estimation using NGOME-LITE

The protein sequences of five S proteins were selected to investigate the presence of deamidation hotspots (SARS-CoV-2, SARS-CoV-URBANI, SARS-CoV-GZ0402, Bat-CoV-RaTG13, and Bat-CoV-WIV1). We used NGOME-LITE (23) to predict deamidation propensity of Asn residues by using predicted t_1/2_ values. To compare sites of deamidation, the protein sequences were aligned using Clustal Omega (58) with default parameters. The residue numbering of SARS-CoV-2 S was used as reference. Conservation of deamidation-prone Asn present in the S protein of SARS-CoV-2 was checked by aligning S sequences from 38 sarbecoviruses (Table S6).

### SARS CoV-2 RBD *Protein expression and purification*

The SARS CoV-2 RBD (GenBank: MN908947) was expressed and purified as reported in (30). The RBD (residues 319–566) was expressed by transient transfection in HEK293-F containing a secretion signal, a C-terminal Sortase motif, and a noncleavable Histidine tag. Transfected cells were incubated at 37 °C with agitation at 220 rpm and 8% CO_2_ atmosphere. Medium containing the secreted protein was harvested 4 days post transfection. During this "production" time, deamidation should occur adding a cumulative amount of deamidated species at zero time. All steps of the purification were performed at 4 °C, during which deamidation rate is expected to be considerably reduced. For RBD purification, an initial immobilized metal ion afﬁnity chromatography step was performed on a His-Trap column (all columns by GE Healthcare) using buffers A (50 mM Tris-HCl, 0.5 M NaCl, 10 mM imidazole, pH 7.4) and B (same as buffer A but with 500 mM imidazole). An additional gel filtration step was performed on a Superdex 200 Increase 10/300 GL column equilibrated with 50 mM Tris-HCl, 150 mM NaCl, pH 7.4. The protein was concentrated to 10 mg/ml and flash frozen in liquid nitrogen and stored at −80 °C until further use.

### hACE2 *Protein expression and purification*

A gene encoding hACE2 residues 1 to 615 followed by a C-terminal HRV 3C cleavage site and a TwinStrepII-tag was cloned in the pXLG expression vector. HEK293F cells transfected with this construct were grown in a TubeSpin bioreactor in FreeStyle293 medium for 72 h at 37 °C with 8% CO_2_ and agitation at 180 rpm. The secreted hACE2 protein was purified from the cell culture medium using a 1-ml StrepTactin Superflow high-capacity cartridge (IBA). After elution, the C-terminal TwinStrepII-tag was removed by cleavage with His6-tagged HRV 3C protease, followed by an immobilized metal ion afﬁnity chromatography step to remove the HRV 3C protease from the sample. Finally, the untagged hACE2 protein was injected into a Superdex200 Increase 10/300 GL size exclusion chromatography column (Cytiva) equilibrated in 50 mM Hepes, pH 7.2, 150 mM NaCl, and 10% glycerol. The protein was concentrated to 1.9 mg/ml, flash-frozen in liquid nitrogen, and stored at −80 °C.

### Chemical biotinylation of hACE2

hACE2 was chemically biotinylated using the EZ-Link NHS-PEG4-Biotin kit (Sigma). The sample was first desalted using a PD-10 gravity flow column (GE Healthcare) in 20 mM sodium phosphate buffer and 150 mM NaCl, pH 7.4. Subsequently, the sample was chemically biotinylated for 2 h on ice, using a 20-fold molar excess of biotin over the target protein. Excess biotin was removed by running the sample through a size exclusion chromatography column (Superdex 200 Increase 10/300 GL). The fractions containing hACE2 were collected and concentrated to approximately 1 mg/ml. A total of 5% v/v glycerol was added before flash-freezing, and the samples were stored at −80 °C until further use.

### Incubation conditions for deamidation rate determination

Purified RBD samples were diluted to 1 mg/ml in buffer 50 mM Tris-HCl, 150 mM NaCl, and 5% v/v glycerol, pH 7.4, filtered with a pore size of 0.22 μm for minimizing bacterial growth, and added in sealed SafeSel vials that were further sealed with parafilm. Samples were incubated at 4 and 37 °C. Independent aliquots were taken at different times for deamidation quantitation.

### GluC and trypsin double digest at low pH for deamidation analysis

Digestion was done at pH 5.7 to avoid extensive deamidation during sample processing. Protein samples were diluted 1:10 in 100 mM ammonium acetate (Fluka), pH 5.7, containing 10% v/v acetonitrile (Fisher Scientific). Disulfide bonds were reduced with 1 mM DTT (Sigma) and 1 mM TCEP (Invitrogen) for 30 min at 37 °C. Proteins were first digested with trypsin (Promega) for 1 h at 37 °C (sample to enzyme ratio 1:20) and then with GluC (Promega) (1:40 ratio) for additional 3 h at 37 °C. The peptides were cleaned up using an OASIS HLB μElution Plate (Waters).

#### Mass spectrometry

An UltiMate 3000 RSLC nano LC system (Dionex) fitted with a trapping cartridge (μ-Precolumn C18 PepMap 100, 5 μm, 300 μm i.d. × 5 mm, 100 Å) and an analytical column (nanoEase M/Z HSS T3 column 75 μm × 250 mm C18, 1.8 μm, 100 Å, Waters) was used, coupled directly to an Orbitrap Fusion Lumos Tribrid Mass Spectrometer (Thermo). Peptide samples were loaded onto the precolumn with a constant flow rate of 30 μl/min for 6 min using 0.05% v/v trifluoroacetic acid in water. Subsequently, peptides were eluted *via* the analytical column (Solvent A: 0.1% formic acid in water) with a constant flow of 0.3 μl/min, with increasing percentage of solvent B (0.1% formic acid in acetonitrile). The peptides were introduced into the Fusion Lumos *via* a Pico-Tip Emitter 360 μm OD × 20 μm ID; 10 μm tip (New Objective) and an applied spray voltage of 2.4 kV. The capillary temperature was set to 275 °C. Full mass scan (MS1) was acquired with a mass range of 375 to 2000 *m/z* in profile mode in the orbitrap with a resolution of 120,000. The filling time was set at a maximum of 50 ms. Data-dependent acquisition was performed with the resolution of the Orbitrap set to 30,000, with a fill time of 86 ms. A normalized collision energy of 34 was applied. MS2 data were acquired in profile mode.

#### Data analysis

Raw data were searched against the Uniprot human (75,069 entries, June 2020) and the SARS-CoV-2 (14 entries, June 2020) reference proteomes using MaxQuant 1.6.14.0 (31). The default parameters were used with the following adjustments: Deamidation of Asn and Gln were set as variable modifications, as well as oxidation of Met and acetylation of protein N termini. No fixed modifications were enabled (only reduction, no alkylation). The minimum peptide length was adjusted to six or seven and the maximum peptide mass to 8000 Da. The precursor mass tolerance of the first search was set to 20 ppm and to 4.5 ppm for the main search. The mass tolerance of fragment ions was set to 20 ppm. For digestion Trypsin/P and GluC were enabled and the missed cleavages were set to 3. Label-free quantification was enabled, with Fast LFQ disabled. IBAQ calculations were enabled. The minimum number of unique peptides was set to 1. A false discovery rate of 1% was applied on both peptide (on modified peptides separately) and protein lists. Decoy mode was set to “revert.” The minimum score for unmodified peptides was 0, for modified peptides 40. The MaxQuant output msms.txt was imported into Skyline 20.2.0.286 (59). Precursor ion chromatograms were extracted, and the values of the peak areas were exported for further calculations. MS1 spectra were inspected manually using Thermo Xcalibur Qual browser 4.2.28.14.

### Biolayer interferometry

The binding of RBD (0 days after purification or 20 days after deamidation at 37 °C) to biotinylated hACE2 was measured by biolayer interferometry using the Octet RED96 system (FortéBio). Concentration-dependent kinetic assays were performed by loading biotinylated hACE2 on streptavidin biosensors (FortéBio), pre-equilibrated in assay buffer (PBS buffer supplemented with 0.1% (w/v) bovine serum albumin and 0.02% (v/v) Tween-20) for 15 min. Prior to the association, a baseline step of 300 s was performed. Subsequently, sensors were dipped in a different well containing 200, 100, 50, 25, and 12.5 nM of RBD for 600 s followed by 900 s of dissociation time in the same buffer. All experiments were carried out at 30 °C. Data were reference-subtracted only for the association and aligned with each other with an in-house Python script, using a 1:1 binding model. All figures were prepared using R and ggplot2. Two independent experiments were done.

## Data availability

All data needed for the assessment or verification of the article's findings is provided as supporting information. The mass spectrometry proteomics data have been deposited to the ProteomeXchange Consortium *via* the PRIDE (60) partner repository with the dataset identifier PXD028071 (peptide: IYQAGSTPCNGVE) Dataset S1 and PXD027873 (peptides: CVNFNFNGLTGTGVLTE and GFNCYFPLQSYGFQPTNGVGYQPYR) Dataset S2.

## Supporting information

This article contains supporting information (8, 9, 24, 25, 26, 37, 61, 62, 63, 64, 65).

## Conflict of interest

The authors declare that they have no conflicts of interest with the contents of this article.
