# Supplementary material for: Deamidation drives molecular aging of the SARS-CoV-2 spike protein receptor-binding motif
Source: J Biol Chem. 2021 Sep 7;297(4):101175. doi: 10.1016/j.jbc.2021.101175 (PMC8421091; doi:10.1016/j.jbc.2021.101175)
Supplement: Figures S1–S4 and Tables S1–S8 [file mmc4.pdf]

RBM Cluster, Hotspots at positions 481, 493 and 501.

```
Pangolin MP789      470  YQAGSTPCNGVEGFNCYFPLQSYGFHPTNGVGYQPYRVVVLSFELLKAPATVCGPKQSTN      528
Pangolin GX-P5E     471  YQAGSTPCNGQVGLNCYYPLERYGFHPTTGVNYQPFRVVVLSFELLNGPATVCGPKLSTT      530
SARS CoV-2 Wuhan    473  YQAGSTPCNGVEGFNCYFPLQSYGFQPTNGVGYQPYRVVVLSFELLHAPATVCGPKKSTN      532
Bat CoV RaTG13      473  YQAGSKPCNGQTGLNCYYPLYRYGFYPTDGVGHQPYRVVVLSFELLNAPATVCGPKKSTN      532
Bat CoV Rs/YN2018A  459  -------------GNGVYTLSTYDFNPNVPVAYQATRVVVLSFELLNAPATVCGPKLSTQ      505
Bat CoV LYRa11      464  FSPDGKPCTP-PAFNCYWPLNDYGFYTTNGIGYQPYRVVVLSFELLNAPATVCGPKLSTD      522
Bat CoV Rs4084      461  YSPGGQSCSA-VGPNCYNPLRPYGFFTTAGVGHQPYRVVVLSFELLNAPATVCGPKLSTD      519
Bat CoV SHC014      461  YSPGGQSCSA-VGPNCYNPLRPYGFFTTAGVGHQPYRVVVLSFELLNAPATVCGPKLSTD      519
Bat CoV WIV1        461  FSPDGKPCTP-PAFNCYWPLNDYGFYITNGIGYQPYRVVVLSFELLNAPATVCGPKLSTD      519
Bat CoV Rs7327      461  FSPDGKPCTP-PAFNCYWPLNDYGFFTTNGIGYQPYRVVVLSFELLNAPATVCGPKLSTD      519
SARS CoV URBANI     460  FSPDGKPCTP-PALNCYWPLNDYGFYTTTGIGYQPYRVVVLSFELLNAPATVCGPKLSTD      518
SARS CoV GZ0402     460  FSPDGKPCTP-PAPNCYWPLNGYGFYTTSGIGYQPYRVVVLSFELLNAPATVCGPKLSTD      518
Bat CoV Rs4231      460  YSPGGQSCSA-IGPNCYNPLRPYGFFTTAGVGHQPYRVVVLSFELLNAPATVCGPKLSTD      518
```

Hotspot 544

```
Pangolin MP789      529  LVKNKCVNFNFNGLTGTGVLTESSKKFLPFQQFGRDIADTTDAVRDPQTLEILDITPCSF      588
Pangolin GX-P5E     531  LVKDKCVNFNFNGLTGTGVLTTSKKQFLPFQQFGRDISDTTDAVRDPQTLEILDITPCSF      590
SARS CoV-2 Wuhan    533  LVKNKCVNFNFNGLTGTGVLTESNKKFLPFQQFGRDIADTTDAVRDPQTLEILDITPCSF      592
Bat CoV RaTG13      533  LVKNKCVNFNFNGLTGTGVLTESNKKFLPFQQFGRDIADTTDAVRDPQTLEILDITPCSF      592
Bat CoV Rs/YN2018A  506  LVKNQCVNFNFNGLKGTGVLTDSSKRFQSFQQFGRDTSDFTDSVRDPQTLEILDITPCSF      565
Bat CoV LYRa11      523  LITNQCVNFNFNGLTGTGVLTPSLKRFQPFQQFGRDFSDFTDSVRDPKTLEVLDISPCSF      582
Bat CoV Rs4084      520  LIKNQCVNFNFNGLTGTGVLTPSSKRFQPFQQFGRDVSDFTDSVRDPKTSEILDISPCSF      579
Bat CoV SHC014      520  LIKNQCVNFNFNGLTGTGVLTPSSKRFQPFQQFGRDVSDFTDSVRDPKTSEILDISPCSF      579
Bat CoV WIV1        520  LIKNQCVNFNFNGLTGTGVLTPSSKRFQPFQQFGRDVSDFTDSVRDPKTSEILDISPCSF      579
Bat CoV Rs7327      520  LIKNQCVNFNFNGLTGTGVLTSSSKRFQPFQQFGRDVSDFTDSVRDPKTSEILDISPCSF      579
SARS CoV URBANI     519  LIKNQCVNFNFNGLTGTGVLTPSSKRFQPFQQFGRDVSDFTDSVRDPKTSEILDISPCSF      578
SARS CoV GZ0402     519  LIKNQCVNFNFNGLTGTGVLTPSSKRFQPFQQFGRDVSDFTDSVRDPKTSEILDISPCSF      578
Bat CoV Rs4231      519  LIKNQCVNFNFNGLTGTGVLTSSSKRFQPFQQFGRDVSDFTDSVRDPKTSEILDISPCSF      578
```

Hotspot 856

```
Pangolin MP789      825  FIKQYGDCLGDIAARDLICAQKFNGLTVLPPLLTDEMIAQYTSALLAGTITSGWTFGAGA      884
Pangolin GX-P5E     827  FIKQYGDCLGDIAARDLICAQKFNGLTVLPPLLTDEMIAQYTSALLAGTITSGWTFGAGA      886
SARS CoV-2 Wuhan    833  FIKQYGDCLGDIAARDLICAQKFNGLTVLPPLLTDEMIAQYTSALLAGTITSGWTFGAGA      892
Bat CoV RaTG13      829  FIKQYGDCLGDIAARDLICAQKFNGLTVLPPLLTDEMIAQYTSALLAGTITSGWTFGAGA      888
Bat CoV Rs/YN2018A  802  FMKQYGECLGDINARDLICAQKFNGLTVLPPLLTDDMIAAYTAALVSGTATAGWTFGAGA      861
Bat CoV LYRa11      819  FMKQYGECLGDISARDLICAQKFNGLTVLPPLLTDEMIAAYTAALVSGTATAGWTFGAGA      878
Bat CoV Rs4084      816  FMKQYGECLGDINARDLICAQKFNGLTVLPPLLTDDMIAAYTAALVSGTATAGWTFGAGA      875
Bat CoV SHC014      816  FMKQYGECLGDINARDLICAQKFNGLTVLPPLLTDDMIAAYTAALVSGTATAGWTFGAGA      875
Bat CoV WIV1        816  FMKQYGECLGDINARDLICAQKFNGLTVLPPLLTDDMIAAYTAALVSGTATAGWTFGAGA      875
Bat CoV Rs7327      816  FMKQYGECLGDINARDLICAQKFNGLTVLPPLLTDDMIAAYTAALVSGTATAGWTFGAGA      875
SARS CoV URBANI     815  FMKQYGECLGDINARDLICAQKFNGLTVLPPLLTDDMIAAYTAALVSGTATAGWTFGAGA      874
SARS CoV GZ0402     815  FMKQYGECLGDINARDLICAQKFNGLTVLPPLLTDDMIAAYSAALVSGTATAGWTFGAGA      874
Bat CoV Rs4231      815  FMKQYGECLGDVNARDLICAQKFNGLTVLPPLLTDDMIAAYTAALVSGTATAGWTFGAGA      874
```

Hotspot 907

```
Pangolin MP789      885  ALQIPFAMQMAYRFNGIGVTQNVLYENQKLIANQFNSAIGKIQDSLSSTASALGKLQDVV      944
Pangolin GX-P5E     887  ALQIPFAMQMAYRFNGIGVTQNVLYENQKLIANQFNSAIGKIQDSLSSTASALGKLQDVV      946
SARS CoV-2 Wuhan    893  ALQIPFAMQMAYRFNGIGVTQNVLYENQKLIANQFNSAIGKIQDSLSSTASALGKLQDVV      952
Bat CoV RaTG13      889  ALQIPFAMQMAYRFNGIGVTQNVLYENQKLIANQFNSAIGKIQDSLSSTASALGKLQDVV      948
Bat CoV Rs/YN2018A  862  ALQIPFAMQMAYRFNGIGVTQNVLYENQKQIANQFNKAISQIQESLTTTSTALGKLQDVV      921
Bat CoV LYRa11      879  ALQIPFAMQMAYRFNGIGVTQNVLYENQKQIANQFNKAISQIQESLTTTSTALGKLQDVV      938
Bat CoV Rs4084      876  ALQIPFAMQMAYRFNGIGVTQNVLYENQKQIANQFNKAISQIQESLTTTSTALGKLQDVV      935
Bat CoV SHC014      876  ALQIPFAMQMAYRFNGIGVTQNVLYENQKQIANQFNKAISQIQESLTTTSTALGKLQDVV      935
Bat CoV WIV1        876  ALQIPFAMQMAYRFNGIGVTQNVLYENQKQIANQFNKAISQIQESLTTTSTALGKLQDVV      935
Bat CoV Rs7327      876  ALQIPFAMQMAYRFNGIGVTQNVLYENQKQIANQFNKAISQIQESLTTTSTALGKLQDVV      935
SARS CoV URBANI     875  ALQIPFAMQMAYRFNGIGVTQNVLYENQKQIANQFNKAISQIQESLTTTSTALGKLQDVV      934
SARS CoV GZ0402     845  ALQIPFAMQMAYRFNGIGVTQNVLYENQKQIANQFNKAISQIQESLTTTSTALGKLQDVV      934
Bat CoV Rs4231      875  ALQIPFAMQMAYRFNGIGVTQNVLYENQKQIANQFNKAISQIQESLTTTSTALGKLQDVV      934
```

Fig S1. Sequence alignments showing conservation of deamidation hotspots.

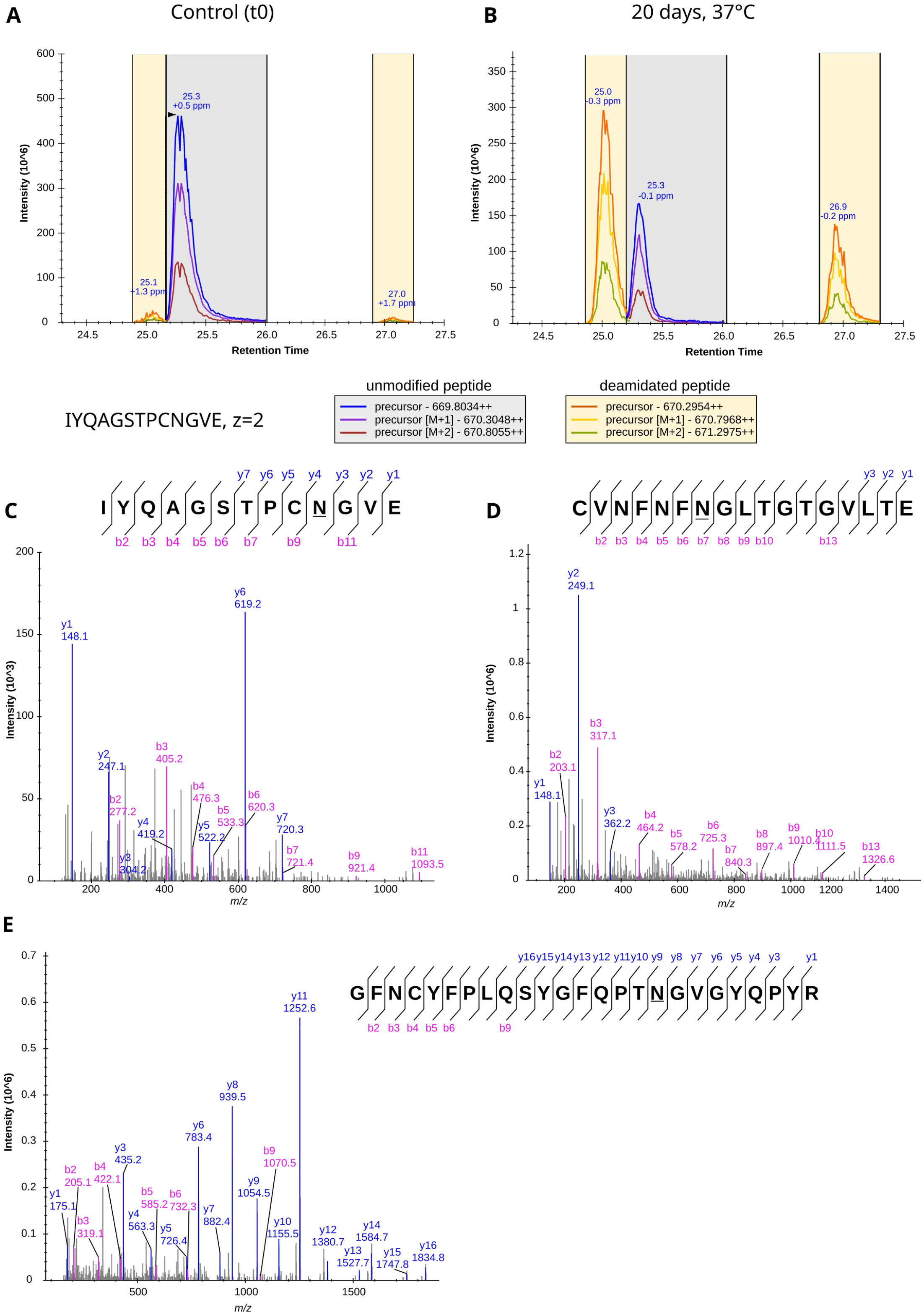

Fig S2. Identification and quantification of deamidated species at the 481, 501 and 544 hotspots.
A) Combined extracted ion chromatograms (XICs) of precursor ions corresponding either to the unmodified (monoisotopic peak at 669.8034, z=2) or deamidated (monoisotopic peak at 670.2954, z=2) peptide IYQAGSTPCNGVE. At t0 only a trace of the deamidated species was observed, eluting at different retention times (RT) as compared to the unmodified peptide. **B**) Combined XIC of the deamidated and unmodified peptides obtained from an aged RBD sample. Two deamidated peptides are observed one eluting at shorter RT and the other at longer RT. **C**) The presence of an aspartic/isoaspartic acid at the position 481 was further confirmed by MS/MS. **D**) Annotated MS/MS spectra showing deamidated species for the peptide CVNFNFNGLTGTGVLTE harboring the hotspot 544. **E**) Annotated MS/MS spectra showing deamidated species for the peptide GFNCYFPLQSYGFQPTNGVGYQPYR harboring the hotspot 501.

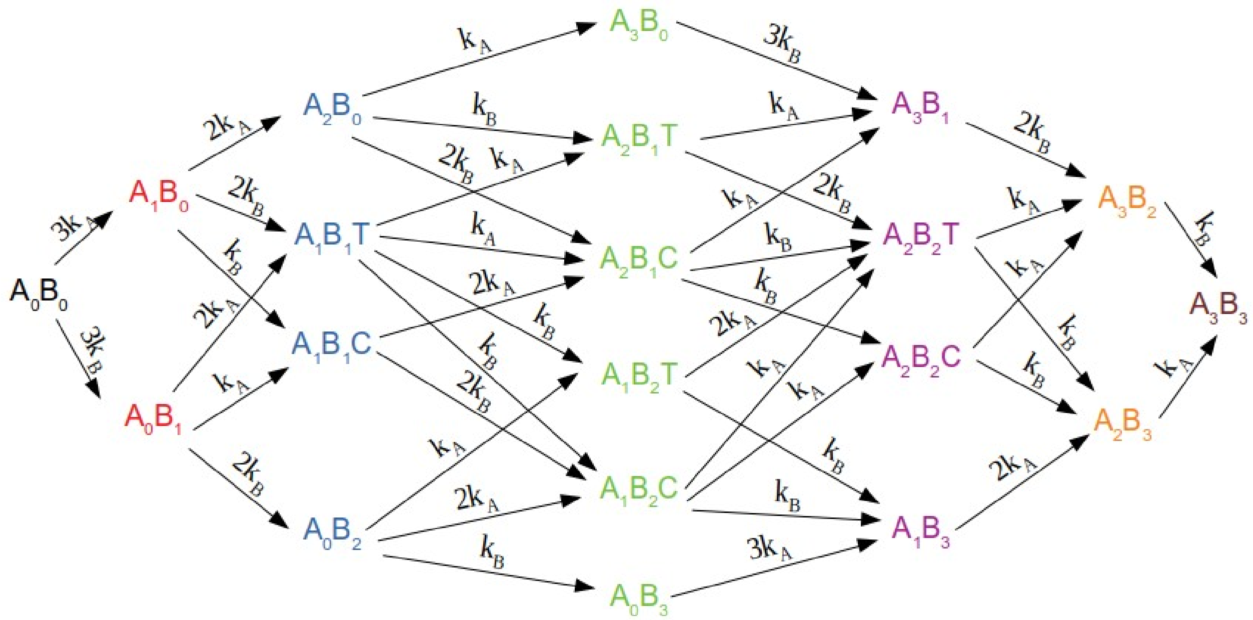

Fig S3. Reaction scheme for deamidation of the S protein trimer.
We considered two deamidation sites in each monomer (A and B) which denote deamidation at Asn 481 and 501, respectively. Both sites deamidate independently with microscopic reaction constants k_A and k_B. We grouped the 2^6 possible deamidation states into 20 species using symmetry considerations. For example, the A_1B_1C species groups trimers where deamidation of one A site and one B site in the trimer took place and the two deamidation events took place in the same monomer (C stands for "cis"). The A_1B_1T species groups trimers where deamidation of one A site and one B site in the trimer took place and the two deamidation events took place in different monomers (T stands for "trans"). The A_2B_0 species groups trimers where deamidation of two A sites and zero B sites in the trimer took place, and the A_0B_2 species groups trimers where deamidation of zero A sites and two B sites in the trimer took place. The 20 trimer species are colored according to the total number of deamidated sites (the color scheme is the same as in Fig 4). The reaction constants for interconversion of the 20 species take degeneracy into account. For example, the species A_0B_0 converts into the species A_1B_0 with a rate constant of $3k_A$ because A_1B_0 groups trimers deamidated at any of the three A sites.

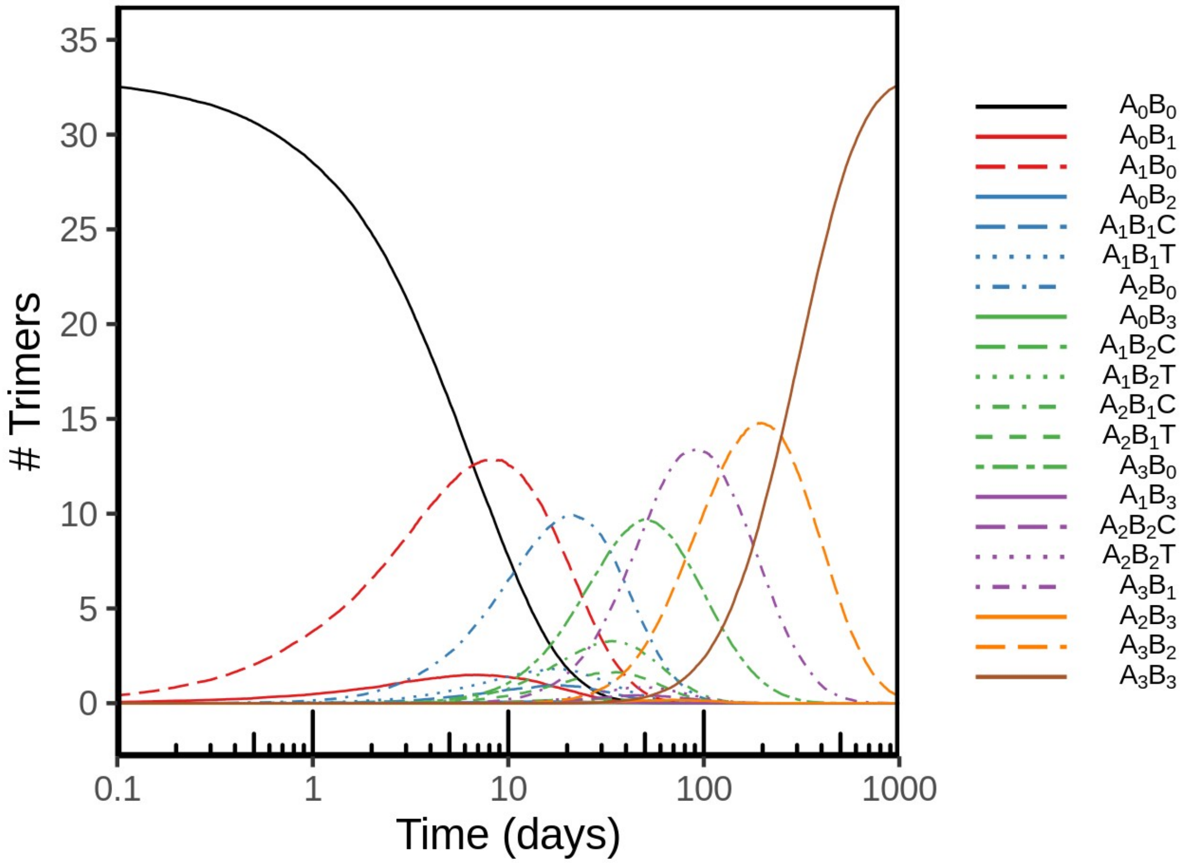

Fig S4. Spike protein hotspot deamidation in the context of the SARS-CoV2 virion at 37 ºC. Simulated time course of the 20 species in Fig S3, using the deamidation half-times from Fig 2 (see Fig S3 for simulation details). A and B denote deamidation at Asn 481 and 501, respectively. We report the average of 1000 simulations using the Gillespie algorithm. The color scheme is the same as in Fig 4.

Table S1. Betacoronaviruses with proven affinity for hACE2. The accession numbers were obtained from GenBank (https://www.ncbi.nlm.nih.gov/genbank)

Virus strain	Accession No.	Host	Reference
SARS-CoV-2 Wuhan-Hu-1	YP_009724390.1	Human<br>2019 outbreak	(1)
SARS-CoV Urbani	AAP13441.1	Human<br>2002 outbreak	(2)
SARS-CoV GZ0402	AY613947.1	Human/Civet<br>2004 outbreak. No secondary infection.	(3)
Bat-CoV WIV1	AGZ48831.1	*Rhinolophus sinicus*	(4, 5)
Bat-CoV RaTG13	QHR63300.2	*Rhinolophus affinis*	(6)

We include the SARS-CoV-2 Wuhan and SARS-CoV Urbani strains that are highly infective viruses with human-to-human transmission, the SARS-CoV GZ0402 strain isolated during the 2003/2004 episode in Guangzhou from a handful of mildly symptomatic individuals with no reported secondary transmission, alongside with human-infecting SARS coronaviruses. We include S proteins from the related Bat SARS-like CoVs RaTG13 (7) and WIV1 (5). The S proteins of SARS-CoV GZ402 share over 98% sequence identity with palm civet SARS-like CoV strains (8, 9), whereas the Bat SARS-like CoVs RaTG13 (7) and WIV1 (5) show 97.6 and 92.3% identity to the SARS CoV-2 and SARS-CoV Spike proteins, respectively.

Table S2. Deamidation half-times ($t_{1/2}$) in days calculated from NGOME-LITE. Positions are according to the SARS-CoV-2 numbering. Glycosylated Asn residues were excluded.

Asn	SARS-CoV-2 Wuhan-Hu-1	SARS-CoV Urbani	SARS-CoV GZ0402	Bat-CoV RaTG13	Bat-CoV WIV1
26	-	-	-	-	449.4
30	227.6	-	-	-	-
46	-	-	-	-	36650.8
74	-	-	-	30.5	-
78	-	-	-	-	1956.9
81	5365.3	11061.3	10851.8	7693.2	13934.6
87	584.5	-	-	634.5	-
99	7772.9	6017.4	5974.4	8012.9	6436.3
111	-	951.4	966.4	-	916.6
121	1042.6	-	-	1042.6	-
125	7366.1	6436.3	6515.9	7366.1	3283.1
132	-	1832.1	1858.6	-	1919.9
137	968.8	-	-	968.8	-
138	-	13302.1	13839.8	-	14607.3
148	1007.9	-	-	1007.9	1266.2
161	-	-	-	-	1021.8
162		621.1	649.1	-	683.5
164	790.3	-	-	790.3	-
176	-	-	-	-	2458.3
185	1787	1866.8	1881.6	1779.6	1583.4
188	14594.1	-		14450.2	-
196	8987.4	1707.3	1707.3	8962.9	1510.7
211	16793.8	-	-	15927.8	-
221	-	1145.2	1130	-	539.8
237	-	11045	10854.3	-	10298
280	1221.9	-	-	1221.9	-
294		9966.9	9966.9		9878.8
317	865	1091.5	1091.5	865	973.8
334	3152.3	3128.7	3110.6	3128.7	3044.4
354	1828.6	-	-	1824.2	-
360	1582.6	1585.8	1562.2	1577.7	1564.3
388	913.2	959.2	947.5	911.6	947.5
394	6749.3	7333.4	7209.2	6804.8	7209.2
422	914	1334.5	1430.3	943.5	1114.3
437	326.8	992.1	8089.9	311.4	783.5
439	868.1	-	-	-	-
440	2731	7035	8263	-	5538.8
448	1827.9	1352.4	1778	1616.8	898.5
450	10014.8	1368.2	1503.5	10778	898.5
460	2606.1	-	-	2731	-
470	-	1680	1521	-	1504.5
481	27	-	-	24.9	-

487	1125.4	892.7	711.1	1136.2	967
493	-	716.8	22.5	-	844.5
501	20.2	-	-	-	31.9
519	-	690.5	690.5	652.2	709.4
532	14947.2	-	-	14594.1	-
536	881.2	6191.5	6191.5	855.4	6191.5
540	1377.4	1353.2	1353.2	1365.2	1353.2
542	1542.3	1513.1	1513.1	1528.7	1513.1
544	27	26.8	26.8	26.8	26.8
556	855.4	-	-	855.4	-
606	897.7	-	-	1011	-
640	-	775.3	822.2	-	484.2
641	5103.9	5647	5883.3	5103.9	3785.2
658	375.5	-	-	384.2	-
679	181.9	-	-	271	-
703	330.5	-	-	351.8	-
710	280.1	883.6	849.6	276.9	849.6
739	-	1507.2	1507.2		1507.2
751	20438.7	20628.1	20628.1	20438.7	20663.4
764	9655.6	9214.1	9286.8	9655.6	9537.5
777	754.1	616.9	685.8	754.1	675.3
824	1561.6	1571.3	1561.6	1561.6	1561.6
845	-	665.5	663.4	-	665.5
856	34.6	34.2	34.2	34.6	34.2
907	33.1	32	32	33.1	32
914	37409.7	33194.1	33194.1	37409.7	33194.1
919	8288.8	6906.7	6906.7	8288.8	6906.7
925	8579.8	7334.7	7334.7	8579.8	7334.7
928	1309.1	3432.1	3432.1	1309.1	3432.1
953	5953	6032.9	6032.9	5953	6032.9
955	2717	2717	2717	2717	2717
960	4047.8	4047.8	4047.8	4047.8	4047.8
969	1709.9	1701.8	1701.8	1709.9	1701.8
978	4531.3	4531.3	4531.3	4531.3	4531.3
1023	2713.3	2713.3	2713.3	2713.3	2713.3
1108	1177.9	1420.4	1420.4	1177.9	1436.5
1119	861	999.9	999.9	883.6	1018.2
1125	1173.7	1284.5	1284.5	-	-
1135	754.1	809.9	809.9	764.2	809.9
1178	5478.6	5478.6	5097.6	5478.6	5478.6
1187	5515.7	5446.4	5071.1	5515.7	5446.4
1192	16532	16163.5	16031.5	16532	16163.5

Table S3. Relative Accessible Surface Area (RASA, Å^2) for deamidation hotspots in SARS-CoV-2 S.

Asn	RASA Close Conformation Probe radius (Å)		RASA Open conformation Probe radius (Å)	
	1.4	3.0	1.4	3.0
481	74.0	70.1	90.9	74.2
501	38.3	25.4	37.9	25.8
544	15.7	0.0	40.8	25.6
856	23.4	2.5	23.8	2.5
907	30.1	2.0	34.5	0.5

Solvent Accessible Surface Area (SASA) and Relative Accessible Surface Area (RASA): SASA was calculated with GETAREA 1.0 (10) using probe radii of 1.4 and 3.0 Å for all residues in S (pdb: 6zgg, (7)) . Side chain SASA is a function of the probe radius and relative normalization was done using the SASA value at each probe radius for a fully exposed Asn residue at position 603. The SASA value for the side chain Asn 603 and a 1.4 Å probe radius is 114.74 Å^2, close to the 114.3 Å^2 reference value reported by (http://curie.utmb.edu/area_man.html) for a Asn residue in a reference tripetide Gly-Asn-Gly in a random coil conformation and calculated as an average of a 30 ensemble conformations.

Table S4. Observed and estimated deamidation half-times for SARS-CoV-2 S hotspots.

	Hotspot				
	481	501	544	856	907
$t_{1/2}$ NGOME-LITE (days)	27.0	20.2	27.0	34.6	33.1
^{18}O-Asp conversion (%) #	4.8	18.9	7.8	4.4	3.2
Experimental $t_{1/2}$ (37 ºC, days)	16.5±3.7	123±23	7.9±1.2	n.d	n.d
Experimental $t_{1/2}$ (4 ºC, days)	362±81	No measurable decay	103±33	n.d	nd
Fold	21.9	-	13.0	-	-
% Unmodified species at t = 0 (4 ºC)	95.5	100	83.2	n.d	n.d
% species with a shorter RT (day 20)	46.6	n.o	68.5	n.d	n.d
% species with longer RT (day 20)	18.5	12.0	14.1	n.d	n.d
Sequence	NGV	NGV	NGL	NGL	NGI

obtained from (11)
n.d; not determined.
n.o; not observed.
RT = Retention time.

Table S5. Quantification of unmodified and deamidated species in the 481, 501 and 544 hotspots. Normalized total area MS1 for unmodified (Unmod) asparagine-containing species, deamidated species eluting with a shorter retention time (Deam SRT) and deamidated species eluting at larger retention times (Deam LRT). Replicates are shown (Exp). n.d, not detected.

Time (days)	Exp	Hotspot 481			Hotspot 501			Hotspot 544		
		IYQAGSTPCNGVE			GFNCYFPLQSYGFQPTNGVGYQPYR			CVNFNFNGLTGTGVLTE		
		t = 4 ˚C								
		Deam SRT	Unmod	Deam LRT	Deam SRT	Unmod	Deam LRT	Deam SRT	Unmod	Deam LRT
0	1	3.13E+08	1.02E+10	1.42E+08	0.00E+00	0.00E+00	0.00E+00	0.00E+00	0.00E+00	0.00E+00
	2	6.13E+08	1.93E+10	2.95E+08	0.00E+00	7.37E+08	0.00E+00	1.29E+08	1.28E+09	4.96E+07
	3	0.00E+00	0.00E+00	0.00E+00	0.00E+00	0.00E+00	0.00E+00	3.44E+08	1.90E+09	1.28E+08
10	1	5.07E+08	9.92E+09	2.25E+08	0.00E+00	7.26E+08	0.00E+00	0.00E+00	0.00E+00	0.00E+00
	2	9.06E+08	1.89E+10	4.55E+08	0.00E+00	3.32E+08	0.00E+00	2.43E+08	1.37E+09	8.92E+07
	3	6.96E+08	1.92E+10	3.04E+08				7.25E+08	2.52E+09	2.33E+08
20	1	8.06E+08	9.47E+09	3.77E+08	0.00E+00	9.18E+08	0.00E+00	0.00E+00	0.00E+00	0.00E+00
	2	1.04E+09	1.86E+10	5.91E+08	0.00E+00	2.94E+08	0.00E+00	3.69E+08	1.33E+09	1.03E+08
	3	8.51E+08	1.89E+10	4.54E+08	0.00E+00	0.00E+00	0.00E+00	0.00E+00	0.00E+00	0.00E+00
30	1	0.00E+00	0.00E+00	0.00E+00	0.00E+00	8.62E+08	0.00E+00	0.00E+00	0.00E+00	0.00E+00
	2	1.41E+09	1.81E+10	7.43E+08	0.00E+00	3.92E+08	0.00E+00	4.94E+08	1.31E+09	1.12E+08
	3	1.12E+09	1.86E+10	5.23E+08	0.00E+00	0.00E+00	0.00E+00	0.00E+00	0.00E+00	0.00E+00
		t = 37 ˚C								
10	1	3.36E+09	5.82E+09	1.47E+09	0.00E+00	0.00E+00	0.00E+00	0.00E+00	0.00E+00	0.00E+00
	2	4.50E+09	1.29E+10	2.83E+09	0.00E+00	8.27E+08	1.03E+08	8.80E+08	4.25E+08	2.02E+08
	3	5.83E+09	1.22E+10	2.18E+09	0.00E+00	2.51E+08	2.19E+07	1.61E+09	7.95E+08	4.49E+08
20	1	5.47E+09	2.72E+09	2.46E+09	0.00E+00	0.00E+00	0.00E+00	0.00E+00	0.00E+00	0.00E+00
	2	1.00E+10	6.21E+09	3.96E+09	0.00E+00	7.27E+08	8.41E+07	7.12E+08	2.21E+08	1.85E+08
	3	7.82E+09	9.82E+09	2.56E+09	0.00E+00	3.49E+08	5.55E+07	1.68E+09	3.46E+08	2.65E+08
30	1	0.00E+00	0.00E+00	0.00E+00	0.00E+00	0.00E+00	0.00E+00	0.00E+00	0.00E+00	0.00E+00
	2	8.04E+09	9.33E+09	2.84E+09	0.00E+00	7.45E+08	1.76E+08	4.17E+08	1.39E+08	1.67E+08
	3	1.06E+10	6.08E+09	3.51E+09	0.00E+00	2.87E+08	5.05E+07	0.00E+00	0.00E+00	0.00E+00

Table S6. Selected *Sarbecoronaviruses*.

	Virus Strain	Accession	Host
1	SARS-CoV-2 Wuhan-Hu-1	YP_009724390.1	Human (2019 outbreak)
2	SARS-CoV Tor2	NC_004718	Human (2002 outbreak)
3	Bat-CoV RaTG13	QHR63300.2	*Rhinolophus affinis*
4	Pangolin-CoV GX-P5E/2017	QIA48641.1	*Manis javanica*
5	Pangolin-CoV MP789/2020	QIG55945.1	*Manis javanica*
6	Bat-CoV LYRa11	AHX37558.1	*Rhinolophus affinis*
7	Bat-CoV ZC45	MG772933	*Rhinolophus pusillus*
8	Bat-CoV ZXC21	MG772934	*Rhinolophus pusillus*
9	Bat-CoV Longquan-140	KF294457	*Rhinolophus monoceros*
10	Bat-CoV HKU3-7	GQ153542	*Rhinolophus sp*
11	Bat-CoV HKU3-3	DQ084200	*Rhinolophus sp*
12	Bat-CoV HKU3-12	GQ153547	*Rhinolophus sp*
13	Bat-CoV 279/2005	DQ648857.1	*Rhinolophus sp*
14	Bat-CoV HuB2013	KJ473814	*Rhinolophus sinicus*
15	Bat-CoV Rp/Shaanxi2011	JX993987	*Rhinolophus pusillus*
16	Bat-CoV JL2012	KJ473811	*Rhinolophus ferrumequinum*
17	Bat-CoV Rf1	DQ412042	*Rhinolophus ferrumequinum*
18	Bat-CoV SX2013	KJ473813	*Rhinolophus ferrumequinum*
19	Bat-CoV Jiyuan-84	KY770860	*Rhinolophus ferrumequinum*
20	Bat-CoV SC2018	MK211374	*Rhinolophus sp*
21	Bat-CoV Yunnan2011	JX993988	*Chaerephon plicata*
22	Bat-CoV GX2013	KJ473815	*Rhinolophus sinicus*
23	Bat-CoV Rp3/2004	DQ071615	*Bat sp.*
24	Bat-CoV Rs4247	KY417148	*Rhinolophus sinicus*
25	Bat-CoV As6526	KY417142	*Aselliscus stoliczkanus*
26	Bat-CoV YN2018C	MK211377	*Rhinolophus affinis*
27	Bat-CoV Rs672/2006	FJ588686	*Rhinolophus sinicus*

28	Bat-CoV Rs4081	KY417143	*Rhinolophus sinicus*
29	Bat-CoV YNLF_31C	KP886808	*Rhinolophus Ferrumequinum*
30	Bat-CoV Rf4092	KY417145	*Rhinilophus ferrumequinum*
31	Bat-CoV Anlong-103	KY770858	*Rhinolophus sinicus*
32	Bat-CoV YN2013	KJ473816	*Rhinolophus sinicus*
33	Bat-CoV Rs4084	KY417144	*Rhinolophus sinicus*
34	Bat-CoV Rs9401	KY417152	*Rhinolophus sinicus*
35	Bat-CoV Rs4874	KY417150	*Rhinolophus sinicus*
36	Civet-CoV 007	AY572034	*Palm Civet*
37	Bat-CoV BtKY72	KY352407	*Rhinolophus sp*
38	Bat-CoV BM48-31/BRG/2008	NC_014470	*Rhinolophus blasii*

Table S7. NGOME-LITE estimated deamidation half-time of hotspots observed in the RBM of *Sarbecoronaviruses*.

Virus Strain	Deamidation $t_{1/2}$ (days)			
	481	487	493	501
Bat-CoV Rs/YN2018A	-	12.3	-	-
Bat-CoV LYRa11	-	-	-	23.25
Bat-CoV WIV1	-	-	-	31.9
Bat-CoV Rs7327	-	-	-	23.75
SARS-CoV Urbani	-	-	-	-
SARS-CoV GZ0402	-	-	22.5	-
Bat-CoV Rs4084	-	-	-	-
Bat-CoV SHC014	-	-	-	-
Bat-CoV Rs4231	-	-	-	-
Pangolin-CoV GX-P5E/2017	26.5	-	-	-
SARS-CoV-2 Wuhan-Hu-1	27	-	-	20.2
Pangolin-CoV MP789/2020	23.08	-	-	17.58
Bat-CoV RaTG13	-	-	-	24.9

Table S8. S protein hotspot deamidation in the context of the SARS-CoV2 virion at 37 ºC. For details see Fig S4 legend

	Time (Days)					
	0	1	2	3	7	14
A_0B_0	33.000 ± 0.000	28.501 ± 2.008	24.789 ± 2.554	21.407 ± 2.662	11.819 ± 2.772	4.348 ± 1.953
A_0B_1	0.000 ± 0.000	0.480 ± 0.696	0.820 ± 0.939	1.137 ± 1.056	1.498 ± 1.146	1.140 ± 1.034
A_0B_2	0.000 ± 0.0000	0.004 ± 0.0632	0.007 ± 0.0834	0.017 ± 0.1293	0.052 ± 0.2221	0.085 ± 0.2790
A_0B_3	0.000 ± 0.0000	0.000 ± 0.0000	0.000 ± 0.0000	0.000 ± 0.0000	0.000 ± 0.0000	0.001 ± 0.0316
A_1B_0	0.000 ± 0.000	3.804 ± 1.876	6.555 ± 2.319	8.713 ± 2.457	12.674 ± 2.771	10.830 ± 2.758
A_1B_1C	0.000 ± 0.000	0.024 ± 0.160	0.071 ± 0.261	0.135 ± 0.365	0.490 ± 0.690	0.867 ± 0.921
A_1B_1T	0.000 ± 0.000	0.035 ± 0.184	0.153 ± 0.392	0.282 ± 0.528	1.004 ± 0.999	1.668 ± 1.256
A_1B_2C	0.000 ± 0.0000	0.000 ± 0.0000	0.001 ± 0.0316	0.004 ± 0.0632	0.033 ± 0.1787	0.136 ± 0.3683
A_1B_2T	0.000 ± 0.0000	0.000 ± 0.0000	0.003 ± 0.0547	0.006 ± 0.0773	0.021 ± 0.1435	0.081 ± 0.2907
A_1B_3	0.000 ± 0.0000	0.000 ± 0.0000	0.000 ± 0.0000	0.000 ± 0.0000	0.000 ± 0.0000	0.004 ± 0.0632
A_2B_0	0.000 ± 0.000	0.146 ± 0.370	0.566 ± 0.734	1.198 ± 1.044	4.302 ± 1.854	8.586 ± 2.541
A_2B_1C	0.000 ± 0.0000	0.003 ± 0.0547	0.013 ± 0.1133	0.031 ± 0.1791	0.360 ± 0.5803	1.479 ± 1.1743
A_2B_1T	0.000 ± 0.0000	0.000 ± 0.0000	0.003 ± 0.0547	0.017 ± 0.1293	0.197 ± 0.4317	0.724 ± 0.8249
A_2B_2C	0.000 ± 0.0000	0.000 ± 0.0000	0.000 ± 0.0000	0.000 ± 0.0000	0.006 ± 0.0773	0.048 ± 0.2185
A_2B_2T	0.000 ± 0.000	0.000 ± 0.000	0.000 ± 0.000	0.000 ± 0.000	0.018 ± 0.133	0.102 ± 0.322
A_2B_3	0.000 ± 0.0000	0.000 ± 0.0000	0.000 ± 0.0000	0.000 ± 0.0000	0.001 ± 0.0316	0.004 ± 0.0632
A_3B_0	0.000 ± 0.0000	0.003 ± 0.0547	0.019 ± 0.1366	0.049 ± 0.2295	0.464 ± 0.6657	2.257 ± 1.4167
A_3B_1	0.000 ± 0.0000	0.000 ± 0.0000	0.000 ± 0.0000	0.004 ± 0.0632	0.060 ± 0.2418	0.599 ± 0.7699
A_3B_2	0.000 ± 0.0000	0.000 ± 0.0000	0.000 ± 0.0000	0.000 ± 0.0000	0.001 ± 0.0316	0.041 ± 0.2034
A_3B_3	0.000 ± 0.000	0.000 ± 0.000	0.000 ± 0.000	0.000 ± 0.000	0.000 ± 0.000	0.000 ± 0.000

References

1. Wu, F., Zhao, S., Yu, B., Chen, Y.-M., Wang, W., Song, Z.-G., Hu, Y., Tao, Z.-W., Tian, J.-H., Pei, Y.-Y., Yuan, M.-L., Zhang, Y.-L., Dai, F.-H., Liu, Y., Wang, Q.-M., Zheng, J.-J., Xu, L., Holmes, E. C., and Zhang, Y.-Z. (2020) A new coronavirus associated with human respiratory disease in China. *Nature*. **579**, 265–269
2. Rota, P. A., Oberste, M. S., Monroe, S. S., Nix, W. A., Campagnoli, R., Icenogle, J. P., Peñaranda, S., Bankamp, B., Maher, K., Chen, M., Tong, S., Tamin, A., Lowe, L., Frace, M., DeRisi, J. L., Chen, Q., Wang, D., Erdman, D. D., Peret, T. C. T., Burns, C., Ksiazek, T. G., Rollin, P. E., Sanchez, A., Liffick, S., Holloway, B., Limor, J., McCaustland, K., Olsen-Rasmussen, M., Fouchier, R., Günther, S., Osterhaus, A. D. M. E., Drosten, C., Pallansch, M. A., Anderson, L. J., and Bellini, W. J. (2003) Characterization of a Novel Coronavirus Associated with Severe Acute Respiratory Syndrome. *Science*. **300**, 1394–1399
3. Liang, G., Chen, Q., Xu, J., Liu, Y., Lim, W., Peiris, J. S. M., Anderson, L. J., Ruan, L., Li, H., Kan, B., Di, B., Cheng, P., Chan, K. H., Erdman, D. D., Gu, S., Yan, X., Liang, W., Zhou, D., Haynes, L. M., Duan, S., Zhang, X., Zheng, H., Gao, Y., Tong, S., Hu, G., Fang, L., Qin, P., and Deng, Y. Laboratory Diagnosis of Four Recent Sporadic Cases of Community-acquired SARS, Guangdong Province, China - Volume 10, Number 10—October 2004 - Emerging Infectious Diseases journal - CDC. 10.3201/eid1010.040445
4. Ge, X.-Y., Li, J.-L., Yang, X.-L., Chmura, A. A., Zhu, G., Epstein, J. H., Mazet, J. K., Hu, B., Zhang, W., Peng, C., Zhang, Y.-J., Luo, C.-M., Tan, B., Wang, N., Zhu, Y., Crameri, G., Zhang, S.-Y., Wang, L.-F., Daszak, P., and Shi, Z.-L. (2013) Isolation and characterization of a bat SARS-like coronavirus that uses the ACE2 receptor. *Nature*. **503**, 535–538
5. Menachery, V. D., Yount, B. L., Sims, A. C., Debbink, K., Agnihothram, S. S., Gralinski, L. E., Graham, R. L., Scobey, T., Plante, J. A., Royal, S. R., Swanstrom, J., Sheahan, T. P., Pickles, R. J., Corti, D., Randell, S. H., Lanzavecchia, A., Marasco, W. A., and Baric, R. S. (2016) SARS-like WIV1-CoV poised for human emergence. *Proc. Natl. Acad. Sci.* **113**, 3048–3053
6. Shang, J., Ye, G., Shi, K., Wan, Y., Luo, C., Aihara, H., Geng, Q., Auerbach, A., and Li, F. (2020) Structural basis of receptor recognition by SARS-CoV-2. *Nature*. **581**, 221–224
7. Wrobel, A. G., Benton, D. J., Xu, P., Roustan, C., Martin, S. R., Rosenthal, P. B., Skehel, J. J., and Gamblin, S. J. (2020) SARS-CoV-2 and bat RaTG13 spike glycoprotein structures inform on virus evolution and furin-cleavage effects. *Nat. Struct. Mol. Biol.* **27**, 763–767
8. Song, H.-D., Tu, C.-C., Zhang, G.-W., Wang, S.-Y., Zheng, K., Lei, L.-C., Chen, Q.-X., Gao, Y.-W., Zhou, H.-Q., Xiang, H., Zheng, H.-J., Chern, S.-W. W., Cheng, F., Pan, C.-M., Xuan, H., Chen, S.-J., Luo, H.-M., Zhou, D.-H., Liu, Y.-F., He, J.-F., Qin, P.-Z., Li, L.-H., Ren, Y.-Q., Liang, W.-J., Yu, Y.-D., Anderson, L., Wang, M., Xu, R.-H., Wu, X.-W., Zheng, H.-Y., Chen, J.-D., Liang, G., Gao, Y., Liao, M., Fang, L., Jiang, L.-Y., Li, H., Chen, F., Di, B., He, L.-J., Lin, J.-Y., Tong, S., Kong, X., Du, L., Hao, P., Tang, H., Bernini, A., Yu, X.-J., Spiga, O., Guo, Z.-M., Pan, H.-Y., He, W.-Z., Manuguerra, J.-C., Fontanet, A., Danchin, A., Niccolai, N., Li, Y.-X., Wu, C.-I., and Zhao, G.-P. (2005) Cross-host evolution of severe acute respiratory syndrome coronavirus in palm civet and human. *Proc. Natl. Acad. Sci.* **102**, 2430–2435
9. Li, W., Zhang, C., Sui, J., Kuhn, J. H., Moore, M. J., Luo, S., Wong, S.-K., Huang, I.-C., Xu, K., Vasilieva, N., Murakami, A., He, Y., Marasco, W. A., Guan, Y., Choe, H., and Farzan, M. (2005) Receptor and viral determinants of SARS-coronavirus adaptation to human ACE2. *EMBO J.* **24**, 1634–1643
10. Fraczkiewicz, R., and Braun, W. (1998) Exact and efficient analytical calculation of the accessible surface areas and their gradients for macromolecules. *J. Comput. Chem.* **19**, 319–333
11. Zhao, P., Praissman, J. L., Grant, O. C., Cai, Y., Xiao, T., Rosenbalm, K. E., Aoki, K., Kellman, B. P., Bridger, R., Barouch, D. H., Brindley, M. A., Lewis, N. E., Tiemeyer, M., Chen, B., Woods, R. J., and Wells, L. (2020) Virus-Receptor Interactions of Glycosylated SARS-CoV-2 Spike and Human ACE2 Receptor. *Cell Host Microbe*. **28**, 586-601.e6
